# Evidence of a turbulent ExB mixing avalanche mechanism of gas breakdown in strongly magnetized systems

**DOI:** 10.1038/s41467-018-05839-5

**Published:** 2018-08-30

**Authors:** Min-Gu Yoo, Jeongwon Lee, Young-Gi Kim, Jayhyun Kim, Francesco Maviglia, Adrianus C. C. Sips, Hyun-Tae Kim, Taik Soo Hahm, Yong-Seok Hwang, Hae June Lee, Yong-Su Na

**Affiliations:** 1 Department of Nuclear Engineering, Seoul National University, Seoul, 08826 Republic of Korea; 2 National Fusion Research Institute, Daejeon, 34133 Republic of Korea; 3 Consorzio CREATE, Univ. Napoli Federico II - DIETI, Napoli, 80125 Italy; 4 JET-EFDA, Culham Science Centre, Abingdon, OX14 3DB UK; 5 European Commission, Brussels, 1049 Belgium; 6 Department of Electrical Engineering, Pusan National University, Busan, 46241 Republic of Korea

## Abstract

Although gas breakdown phenomena have been intensively studied over 100 years, the breakdown mechanism in a strongly magnetized system, such as tokamak, has been still obscured due to complex electromagnetic topologies. There has been a widespread misconception that the conventional breakdown model of the unmagnetized system can be directly applied to the strongly magnetized system. However, we found clear evidence that existing theories cannot explain the experimental results. Here, we demonstrate the underlying mechanism of gas breakdown in tokamaks, a turbulent ExB mixing avalanche, which systematically considers multi-dimensional plasma dynamics in the complex electromagnetic topology. This mechanism clearly elucidates the experiments by identifying crucial roles of self-electric fields produced by space-charge that decrease the plasma density growth rate and cause a dominant transport via ExB drifts. A comprehensive understanding of plasma dynamics in complex electromagnetic topology provides general design strategy for robust breakdown scenarios in a tokamak fusion reactor.

## Abstract (web summary)

Gas breakdown mechanism in plasma under the influence of complex electromagnetic field topology is still debatable. Here the authors present the evidence of the E×B mixing avalanche for gas breakdown in magnetized plasmas in fusion devices as tokamak.

## Introduction

The gas breakdown, such as spark and lightning, has fascinated scientists for a long time. A remarkable Townsend’s study^1^ around 1900 revealed a fundamental principle of the electron avalanche, but it cannot describe many discharges observed in nature and laboratory^2^. Breakthroughs in the gas breakdown physics came with the consideration of the self-electric fields produced by plasma space-charge, which the Townsend theory ignored. Based on this, different types of electrical breakdown, such as streamer^3–5^ and leader breakdown^6^, have been well identified and classified for unmagnetized systems.

However, these conventional gas breakdown models of unmagnetized systems cannot be directly extended to strongly magnetized systems, because the gyro-motion and the guiding center motion of the charged particles newly come into play. This is especially true for tokamak, known as a promising candidate for magnetic confinement fusion reactor, which has strong magnetic fields (>1T) within toroidal periodic geometry. The electric gas breakdown in the tokamak, called the ohmic breakdown^7,8^, has distinct operation conditions of extremely low electric fields (~1 V m^−1^), gas pressure (~mPa), and very long magnetic field lines (>1000 m). Although the ohmic breakdown has generally been used over 50 years, its physical mechanism has been still obscured due to the complex electromagnetic (EM) structures and a lack of experimental data.

Previous studies^9–14^ have simply adopted the traditional Townsend theory of the unmagnetized system by assuming that the self-electric fields are negligible and the guiding center of charged particles always follows the magnetic field line. This misconception still persists, even though the existence of strong self-electric fields (~kV m^−1^) in the RZ plane and related ExB drift motion during breakdown was observed 30 years ago^15^. For example, the Townsend theory-based methodologies neglecting the self-electric fields, such as empirical condition^16^ and field-line-following analysis^17–20^, are still being widely used to estimate the breakdown scenario. However, we have revealed that the Townsend theory or any existing theory cannot explain mysterious experimental results such as slow plasma formation and homogeneous plasma structure along magnetic field line^21,22^. Therefore, a systematic gas breakdown model is required to understand these complex avalanche phenomena in the strongly magnetized system.

Here, we present a systematic study on gas breakdown mechanism in the strongly magnetized system by identifying significant roles of the self-electric fields in the parallel and perpendicular direction to the magnetic fields, respectively. A toroidally symmetric plasma model provides a comprehensive understanding of the breakdown physics in the RZ plane with critical plasma densities. Above the critical densities, the self-electric fields drastically reduce the avalanche growth rate and cause a dominant perpendicular transport via ExB drift motion (see Supplementary Movie 1 for an overview). A multi-dimensional particle simulation with BREAK^23^, clearly demonstrates these physical mechanisms by reproducing the experimental results of the KSTAR^24^ device. We also suggest a powerful topology analysis method to predict the location of the plasma formation in the complex EM structure that greatly facilitates the design of robust and optimized breakdown scenarios for ITER^25^ and future reactors, which is essential to initiate fusion plasmas.

## Results

### Experimental evidence contradictory to existing theories

In the absence of the magnetic fields, the electron avalanche under constant-like external electric fields can be described by the Townsend theory or the streamer theory depending on the operation condition. The Townsend theory describes normal electron avalanche in the operation regime of low *pd*, where *p* is the gas pressure and *d* is the gap length between electrodes. The streamer theory has been developed to explain much faster avalanche in the higher *pd* operation. This fast avalanche is due to the self-electric fields of a highly amplified single avalanche, known as Meek breakdown condition^5^, exp(α*d*) ≥ 10^8^, where α is the first Townsend coefficient.

**Fig. 1:**
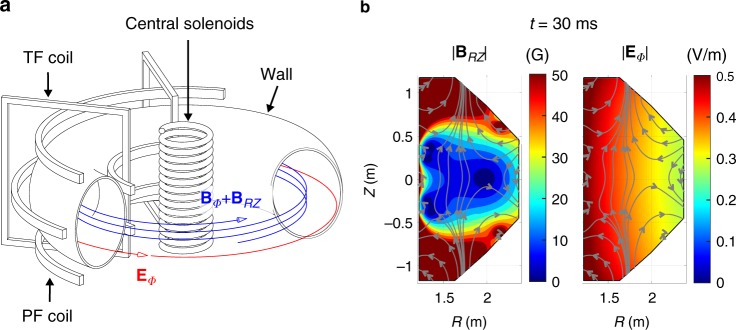
Schematic diagram of ohmic breakdown. **a** The tokamak device consists of a donut-shaped axisymmetric wall and several coil segments. TF (Toroidal Field) coils produce strong static toroidal magnetic fields (**B**_*ϕ*_), and central solenoids swing their currents to induce the toroidal electric fields (**E**_*ϕ*_) by Faraday’s law to make the electron avalanche. The toroidal electric fields induce eddy currents in the wall, the eddy currents and central solenoids' currents produce undesirable stray vertical magnetic fields (**B**_*RZ*_) in the RZ plane. The time-varying current waveforms of the PF (Poloidal Field) coils are designed to provide vertical magnetic fields that cancel out the stray magnetic fields effectively. As a result, very weak vertical magnetic fields (|**B**_*RZ*_|~10^−3^ T) and strong toroidal magnetic fields (|**B**_*ϕ*_ | ~ 1 T) comprise helical magnetic fields (**B**_*ϕ*_ + **B**_*RZ*_) of the complex structure. The helical fields have very small pitch angles (*θ*_*B*_~10^−3^ rads) and open to the walls with very long connections lengths (~10^3^ m). **b** A snapshot of the complex electromagnetic (EM) structures in the RZ plane in a KSTAR reference breakdown scenario at *t* = 30 ms. Gray lines and arrows indicate the magnetic field lines. Color contours show the magnitudes of (left) **B**_*RZ*_ in Gauss unit (1 G = 10^−4^ T) and (right) **E**_*ϕ*_ in V m^−1^ unit. The EM structures are strongly inhomogeneous and change dynamically during the ohmic breakdown (see Supplementary Fig. 1 for more details)

**Fig. 2:**
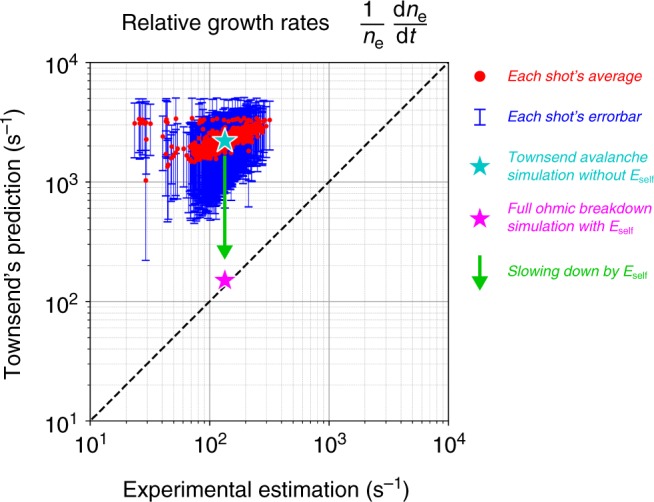
Relative growth rates of plasmas in experiments and simulations. The abscissa is the experimental estimation of the relative growth rate, and the ordinate is the Townsend theory’s prediction on the relative growth rate for 948 shots of KSTAR ohmic breakdown experiments. The experimental relative growth rate of the plasma density ($\frac{1}{{\it{n}_{\mathrm{e}}}}\frac{{\mathrm{d}\it{n}_{\mathrm{e}}}}{{\mathrm{d}\it{t}}}$) can be estimated from the growth rate of measured plasma currents. The Townsend’s prediction on the relative growth rate of each shot is calculated using certain ranges of values for experimental electric fields, neutral gas pressure, and pre-assumed connection length due to the uncertainties of the diagnostics (see Methods for details). The red dot and the blue error bar indicate the average and standard deviation of Townsend’s predictions for each ohmic breakdown shot of the KSTAR device, respectively. The relative growth rates of the Townsend’s prediction are 1–2 orders of magnitude larger than that of the experimental estimation. The two relative growth rates are calculated from two different BREAK simulations (Fig. 6a) to compare with the experiments. The cyan star represents the relative growth rates of the Townsend avalanche simulation without considering the self-electric fields, and the magenta star represents that of the full ohmic breakdown simulation with considering the self-electric fields. The green arrow indicates a significant slowing down of the growth rate due to the self-electric fields, which agrees with the experiments quantitatively

Interestingly, the ohmic breakdown in the tokamak (Fig. 1, Supplementary Fig. 1) seems to satisfy the criteria of both theories: low *pd* (~1 Pa m) and high amplification of the single avalanche (exp(α*d*) ≥ 10^8^). However, we found that none of both theories can explain mysterious experimental results of the ohmic breakdown (Supplementary Fig. 2). First, the estimated avalanche growth rate in experiments is 1–2 orders of magnitudes slower than these theories (Fig. 2). Second, a sustainable homogeneous plasma structure observed along the magnetic field lines (Supplementary Figs. 2e and 3e) cannot be explained by the Townsend theory, because the Townsend avalanche should exhibit a profile of exponential growth along the magnetic field line, which results in a sharply localized structure at the avalanche head (Supplementary Figs. 3b, c, and 4a). The streamer theory also cannot describe the sustainable homogeneous structure, because the streamers should disappear soon after presenting zigzag and multiple branch structures (Supplementary Fig. 4b).

It is noteworthy to compare the electron avalanche with the snow avalanche to understand why the existing theories cannot explain the ohmic breakdown experiments. The traditional understanding of the electron avalanche under the strong magnetic fields is the same as the principle of the snow avalanche. The electrons (snowballs) are swept by the external electric fields (gravity) along the magnetic field lines (mountain slopes) (Supplementary Fig. 3a). As a result of this one-way parallel transport (downward snow flow) with the ionization reactions (snow accumulation), the electron density (snow height) at the avalanche head (bottom of the mountain) should be greatly higher than that of the avalanche tail (mountaintop) (Supplementary Fig. 3f). However, unlike this traditional understanding, the ohmic breakdown experiments show the homogeneous plasma structure along the magnetic field line in which the electron density is uniform from the avalanche tail to the avalanche head (Supplementary Fig. 3g). This phenomenon implies that the underlying transport mechanism of the ohmic breakdown plasma is entirely different from the one-way transport induced by the external electric fields. Therefore, it is essential to figure out what transport is newly activated during the ohmic breakdown to understand the sustainable homogeneous plasma structure along the magnetic field lines.

### Modeling of multi-dimensional plasma dynamics

We deduced that the existing avalanche theories of the unmagnetized system cannot be directly extended to the strongly magnetized system, because they do not consider the nature of gyro-motion and guiding center motion of the charged particles in the magnetic fields. The plasma physics in the parallel direction to the magnetic field could be similar to that of an unmagnetized plasma, but the perpendicular direction to the magnetic field is entirely different. The perpendicular dynamics could significantly influence the avalanche phenomena in the magnetized system. Therefore, a systematic gas breakdown theory should be able to treat the perpendicular dynamics, as well as the parallel dynamics.

**Fig. 3:**
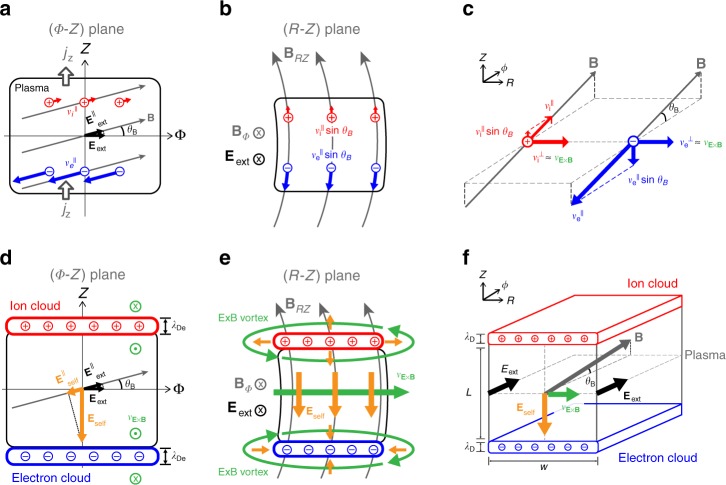
Toroidally symmetric plasma model. Schematic diagrams of toroidally symmetric plasma model in *ϕ*-Z plane view (**a** and **d**), R-Z plane view (**b**, **e**), and three-dimensional view (**c**, **f**). **a**, **b** represent the guiding center motion of ions (red) and electrons (blue) in the low-density plasma ($n \ll n_{{\mathrm{crit}},\parallel }$, $n \ll n_{{\mathrm{crit}}, \bot }$) where the self-electric field effects are negligible. **c** to **f** depicts the self-electric fields (orange) and related ExB transports (green) in the high-density plasma ($n \ge n_{{\mathrm{crit}},\parallel }$, $n \ge n_{{\mathrm{crit}}, \bot }$). The magnitude of the magnetic field pitch angle *θ*_*B*_ in the order of 10^−3^ radians is exaggerated in the figure for clarity

For this purpose, we established a simplified theoretical model to study the general characteristics of gas breakdown physics by focusing on a topological relationship between the self-electric fields and strong magnetic fields. The toroidal periodic geometry and toroidally symmetric external electromagnetic fields in the tokamak cause the physical parameters of plasma, such as density and temperature, to become toroidally symmetric. The toroidally symmetric mode (*n* = 0) is the most dominant one because the average of any sinusoidal fluctuation in the toroidal direction (*n* ≥ 1) is zero due to the toroidal periodicity, where *n* is a toroidal mode number. Therefore, a toroidally symmetric plasma model (Fig. 3) is beneficial to study the roles of self-electric fields and related plasma transports regarding the strong magnetic fields by neglecting high-order toroidal fluctuations. This model can provide a clear and comprehensive understanding of breakdown physics that comprises of the parallel and the perpendicular dynamics in the RZ plane. We also developed a multi-dimensional particle simulation code BREAK to verify the theoretical model and to systematically investigate dynamic plasma evolutions in the realistic complex electromagnetic structures. BREAK is applied to simple artificial scenarios and a reference ohmic breakdown scenario of KSTAR^26^ (see Methods).

### Ohmic heating power drop as a parallel plasma response

**Fig. 4:**
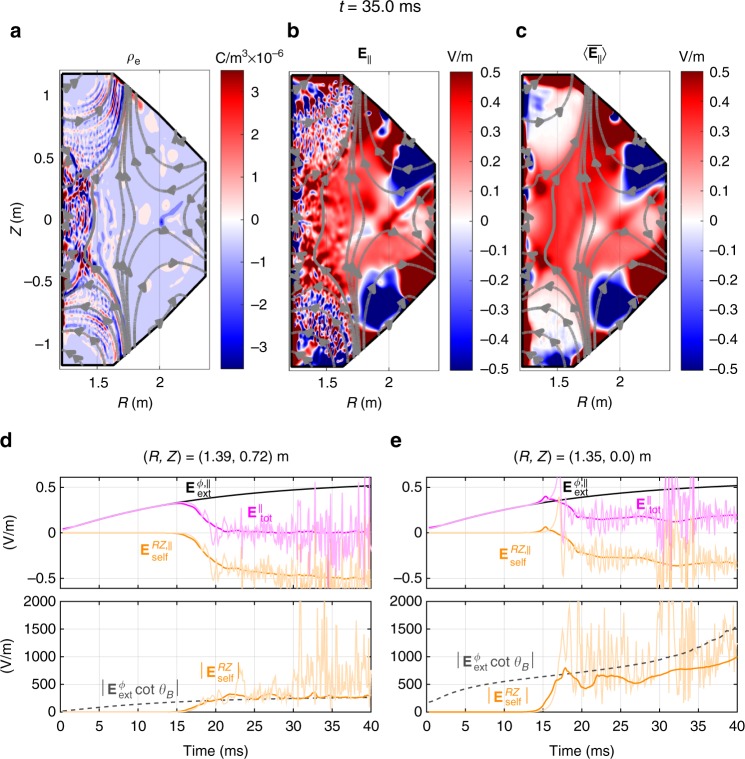
Fluctuating electric field and its temporal average. **a**, **b** show snapshots at *t* = 35 ms for fluctuating charge densities and parallel component of the total electric fields, respectively. **c** is a snapshot of the temporal average of the fluctuating total electric fields. **d**, **e** show temporal evolutions of the electric fields at two different points, respectively. Upper panels show the parallel components of external (black), self (orange), and total (magenta) electric field, and lower panels show the magnitudes of the self-electric fields in the RZ plane (orange) and their theoretical predictions in the ideal condition (Eq. (2), dashed gray). Thin and transparent sold lines are fluctuating electric fields measured in every 100 μs, and thick and opaque lines are the mean electric fields averaged over 3 ms

**Fig. 5:**
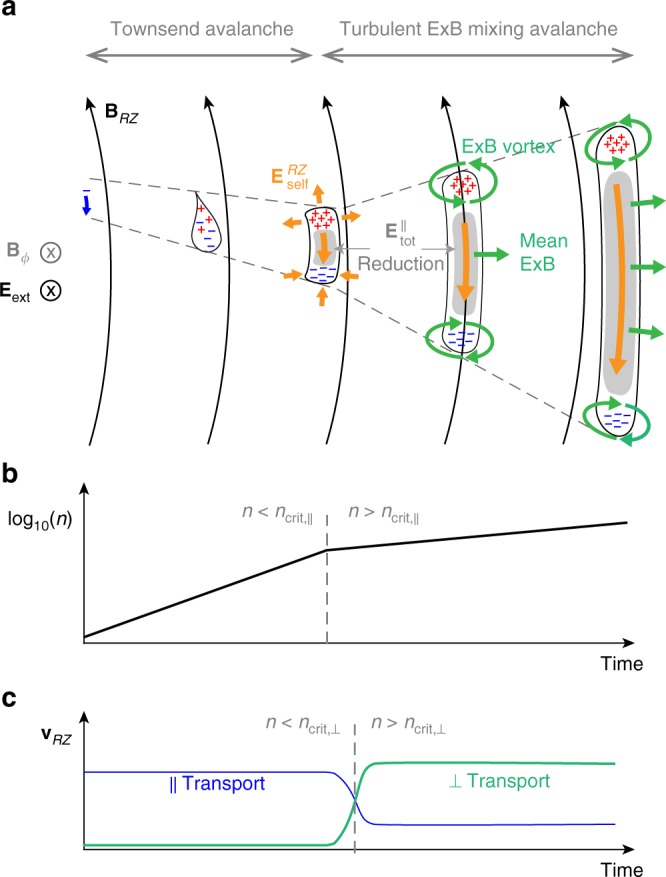
Schematic diagram of turbulent ExB mixing avalanche. **a** Temporal plasma evolution in the RZ plane. **b** Temporal evolution of the plasma density in log-scale regarding the critical parallel density. **c** Temporal evolution of plasma transports in the RZ plane regarding the perpendicular critical density. The initial stage of the ohmic breakdown is the same as the Townsend avalanche that seed electrons move parallel to and increase exponentially along the magnetic field lines. When the plasma density exceeds critical densities ($\it{n}_{{\mathrm{crit}},\parallel }$ and $\it{n}_{{\mathrm{crit}}, \bot }$), the breakdown mechanism changes to the turbulent ExB mixing avalanche due to the self-electric fields ($E_{{\mathrm{self}}}^{RZ}$) produced by the plasma. The plasma growth rate decreases significantly and the enhanced ExB transports dominate the whole plasma transport. The faster turbulent ExB mixing rapidly diffuses the plasma along the magnetic field line, and slower mean ExB moves the plasma across the field lines

The roles of electric fields can be understood in the parallel and the perpendicular dynamics with respect to the magnetic fields. We first focus on the parallel dynamics that the parallel component of the electric field heats the electrons and determines the electron temperature and the avalanche growth rate. This parallel electric field plays the same role as the electric field in the unmagnetized system. The toroidally symmetric plasma model (Fig. 3) describes the relationships between the external electric fields ${\mathbf{E}}_{{\mathrm{ext}}}^\phi$, self-electric fields ${\mathbf{E}}_{{\mathrm{self}}}^{RZ}$, and the pitch angle of the magnetic fields *θ*_*B*_. When ${\mathbf{E}}_{{\mathrm{ext}}}^\phi$ is applied to the plasma, the electrons and ions are separated in the RZ plane along the magnetic field lines (Fig. 3a, b). The resulting toroidally symmetric space-charges produce self-electric fields in the RZ plane, ${\mathbf{E}}_{{\mathrm{self}}}^{RZ}$, to attract each other (Fig. 3d–f). The attracting force by the parallel self-electric fields $E_{{\mathrm{self}}}^{RZ,\parallel } \equiv {\mathbf{E}}_{{\mathrm{self}}}^{RZ} \cdot {\hat{\mathbf b}}$, competes against the separating force by parallel external electric fields $E_{{\mathrm{ext}}}^{\phi ,\parallel } \equiv {\mathbf{E}}_{{\mathrm{ext}}}^\phi \cdot {\hat{\mathbf b}}$, where ${\hat{\mathbf b}}$ is the unit vector of magnetic fields. To generate sufficient attracting force comparable to the separating force ($\left| {E_{{\mathrm{ext}}}^{\phi ,\parallel }} \right| \approx \left| {E_{{\mathrm{self}}}^{RZ,\parallel }} \right|$), the self-electric fields must be 2–3 orders of magnitudes larger than the external electric fields ($\left| {{\mathbf{E}}_{{\mathrm{self}}}^{RZ}} \right| \approx \left| {{\mathbf{E}}_{{\mathrm{ext}}}^\phi } \right|\cot \theta _B \gg \left| {{\mathbf{E}}_{{\mathrm{ext}}}^\phi } \right|$) due to the very small pitch angle (*θ*_*B*_~10^−3^ rads). It is noteworthy that this expression for the self-electric field was first suggested in ref. ^15^ and found in a good agreement with probe measurements. The external electric fields dominate in the initial phase of the breakdown because very low space-charge in the initial plasma cannot make the sufficiently large self-electric fields. However, as the plasma density exponentially increases with the electron avalanche, $E_{{\mathrm{self}}}^{RZ,\parallel }$ also increases to be able to cancel $E_{{\mathrm{ext}}}^{\phi ,\parallel }$. We found that the short charge separation within the Debye length scale can yield a sufficiently large $E_{{\mathrm{self}}}^{RZ,\parallel }$ to cancel out $E_{{\mathrm{ext}}}^{\phi ,\parallel }$ (Fig. 3d, f) when the plasma density exceeds a critical parallel density *n*_crit,||_:

1 $$n_{{\mathrm{crit}},\parallel } \equiv \left( {{{\epsilon }}_0/kT_{\mathrm{e}}} \right)\cot ^2\left( {\theta _B} \right)\left( {E_{{\mathrm{ext}}}^\phi } \right)^2\gamma ^{ - 2}$$

where *ε*_0_ is the permittivity, *k* is the Boltzmann constant, *T*_e_ is the electron temperature, and *γ* is a shape factor of the plasma (see Methods for details). Ideally, the total magnitude of the parallel electric fields decreases drastically to zero: $E_{{\mathrm{tot}}}^\parallel = E_{{\mathrm{ext}}}^{\phi ,\parallel } + E_{{\mathrm{self}}}^{RZ,\parallel } \approx 0$. However, in real situations, $E_{{\mathrm{self}}}^{RZ,\parallel }$ cannot cancel $E_{{\mathrm{ext}}}^{\phi ,\parallel }$ out at everywhere simultaneously because the self-electric fields must have various directions and magnitudes to satisfy the Gauss’s law ($\nabla \cdot {\mathbf{E}} = \rho /{\it{\epsilon }}_0$) for the fluctuating charge densities (*ρ*) in the device (Fig. 4a). At a specific time, $E_{{\mathrm{self}}}^{RZ,\parallel }$ not only cancels $E_{{\mathrm{ext}}}^{\phi ,\parallel }$ out at some positions but also strengthens $E_{{\mathrm{ext}}}^{\phi ,\parallel }$ at the other positions (Fig. 4b). Interestingly, although the self-electric fields rapidly fluctuate in the order of microseconds (Supplementary Movie 2 and 3), they tend to cancel out the external fields in the average of millisecond time scale as the plasma response. A mean self-electric field $\langle \overline {{\mathbf{E}}_{{\mathrm{self}}}^{RZ}} \rangle$, which is a temporal average of the fluctuating self-electric field, effectively cancel out $E_{{\mathrm{ext}}}^{\phi ,\parallel }$ at overall plasma region (Fig. 4c and Supplementary Movie 2) via its parallel component $\langle \overline {{\mathbf{E}}_{{\mathrm{self}}}^{RZ}} \rangle^\parallel \equiv \langle \overline {{\mathbf{E}}_{{\mathrm{self}}}^{RZ}} \rangle \cdot {\hat{\mathbf b}}$. $\langle \overline {{\mathbf{E}}_{{\mathrm{self}}}^{RZ}} \rangle$ tends to be tangential to **B**_*RZ*_ (Fig. 5a) and its maximum magnitude is $\left| {{\mathbf{E}}_{{\mathrm{ext}}}^\phi } \right|\cot\theta _B$ (Fig. 4d, e) as follows:

2 $$\langle \overline {{\mathbf{E}}_{{\mathrm{self}}}^{RZ}}\rangle = \left| {{\mathbf{E}}_{{\mathrm{ext}}}^\phi } \right|\cot\theta _B\left( { - {\hat{\mathbf E}}_{{\mathrm{ext}}}^\phi \cdot {\hat{\mathbf B}}_\phi } \right){\hat{\mathbf B}}_{RZ}$$

where ${\hat{\mathbf E}}_{{\mathrm{ext}}}^\phi$, ${\hat{\mathbf B}}_\phi$, and ${\hat{\mathbf B}}_{RZ}$ are the unit vectors of toroidal electric fields, toroidal magnetic fields, and poloidal magnetic fields, respectively. Notably, this reduction of the total parallel electric field decreases the ohmic heating power to the electrons, $P_{{\mathrm{ohm}},{\mathrm{e}}} = q_{\mathrm{e}}n_e{\mathbf{E}}_{\mathrm{tot}}^\parallel \cdot {\mathbf{v}}_{\mathrm{e}}^\parallel$, where *q*_e_ is the electron charge, *n*_e_ is the electron density, and ${\mathbf{v}}_{\mathrm{e}}^\parallel$ is the parallel electron velocity. This ohmic heating power drop reduces the electron temperature and the ionization reactions and hence causes a slowing down of the avalanche growth rate (Fig. 5b). Therefore, the ohmic heating power drop as a result of the parallel plasma response is one of the key mechanisms underlying the mysterious slow plasma formation of the ohmic breakdown.

**Fig. 6:**
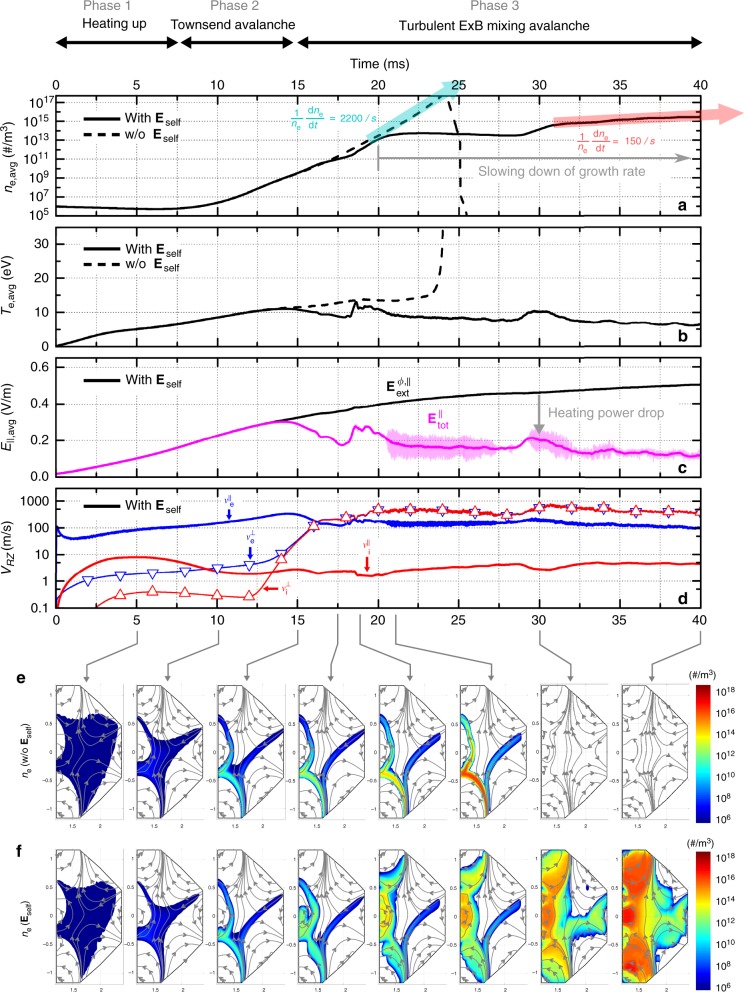
Particle simulation results of the KSTAR reference breakdown scenario. Two different cases that with (solid) and without (dash) considering the self-electric fields are simulated using the BREAK code for the same initial and boundary conditions. The average electron density and temperature for these two cases are compared in **a**, **b**, respectively. The transparent block arrows in **a** represent the relative growth rates of the plasma densities for the two cases. **c**, **d** present more detailed results of the case that considers the self-electric fields. **c** presents the average magnitudes of parallel components of the external electric fields (black) and total electric fields (magenta). **d** presents the average speeds of the parallel (solid line without symbols) and the perpendicular (solid line with triangle symbols) transports projected onto the RZ plane for electrons (blue) and ions (red). Two-dimensional snapshots of the electron densities of the two cases are compared in **e**, **f** (see Supplementary Movie 1, 2 and 4 for details)

The BREAK simulations well capture this slow plasma formation due to the self-electric fields. In the case of a Townsend avalanche simulation without considering the self-electric fields, the plasma density increases exponentially resulting in an unrealistic full ionization of the neutral gas within a short time (Fig. 6a, b, e). In contrast, in the case of a full ohmic breakdown simulation by considering the self-electric fields, the parallel plasma dynamics significantly change when the plasma density exceeds *n*_crit,||_ (Fig. 6a and Supplementary Fig. 10a). The averaged $E_{\mathrm{tot}}^\parallel$ and the ohmic heating power decrease because $\langle \overline {{\mathbf{E}}_{{\mathrm{self}}}^{RZ}} \rangle^\parallel$ partially cancels $E_{{\mathrm{ext}}}^{\phi ,\parallel }$ depending on the plasma shape and location (Fig. 6b, c, and Supplementary Fig. 10b, c). The resulting lower electron temperature causes the slower plasma growth rate and delays the breakdown time (Fig. 6a and Supplementary Movie 1), which agrees well with the experimental findings. We would like to emphasize that the relative growth rates of the plasma ($\frac{1}{n}\frac{{\mathrm{d}\it{n}}}{{\mathrm{d}\it{t}}}$) calculated from the two different simulation cases quantitatively agree with the experiments (Figs. 2, 6a).

### Dominant ExB transports as perpendicular plasma responses

The perpendicular component of the electric field does not change the energy of the charged particle, but it induces a drift motion of the guiding center in the perpendicular direction to the magnetic fields, known as ExB drift **v**_E×B_ = (**E** × **B)**/*B*^2^ where *B* is the magnitude of the magnetic field. This ExB transport has been paid very little attention in the ohmic breakdown physics, since previous researchers have focused only on the external electric field that is too small to induce considerable ExB drift. In that sense, for several decades, the parallel electron transport $v_{\mathrm{e}}^\parallel$ and ion sound speed $C_{\mathrm{s}}^\parallel = \sqrt {kT_{\mathrm{e}}/m_{\mathrm{i}}}$ have been widely believed to be a dominant transport mechanism during the ohmic breakdown and the consecutive start-up phase, respectively. The perpendicular electron transports, $v_{\mathrm{e}}^ \bot$, and all ion transports, $v_{\mathrm{i}}^\parallel$ and $v_{\mathrm{i}}^ \bot$, have been ignored.

However, we realized that the plasma transport mechanisms change dramatically when the self-electric fields come into play. As described in the previous section, the plasma tends to produce the strong ${\mathbf{E}}_{{\mathrm{self}}}^{RZ}$ to cancel $E_{{\mathrm{ext}}}^{\phi ,\parallel }$ as the plasma response. This strong ${\mathbf{E}}_{{\mathrm{self}}}^{RZ}$ with the toroidal magnetic field **B**_ϕ_ induces a fast ExB drift motion in the RZ plane ${\mathbf{v}}_{{\mathrm{E}} \times {\mathrm{B}}}^{RZ} = \left( {{\mathbf{E}}_{{\mathrm{self}}}^{RZ} \times {\mathbf{B}}_\phi } \right)/B^2$. Although ${\mathbf{E}}_{{\mathrm{self}}}^{RZ}$ could also induce a slow ExB drift motion into the toroidal direction (${\mathbf{v}}_{{\mathrm{E}} \times {\mathrm{B}}}^\phi = \left( {{\mathbf{E}}_{{\mathrm{self}}}^{RZ} \times {\mathbf{B}}_{RZ}} \right)/B^2$)  by interacting with the small poloidal magnetic fields **B**_*RZ*_, it does not play important roles during the ohmic breakdown due to its small magnitude ($\left| {{\mathbf{v}}_{{\mathrm{E}} \times {\mathrm{B}}}^\phi } \right|/\left| {{\mathbf{v}}_{{\mathrm{E}} \times {\mathrm{B}}}^{RZ}} \right| \le \left| {{\mathbf{B}}_{RZ}} \right|/\left| {{\mathbf{B}}_\phi } \right| \ll 1$) and the toroidal symmetry of the plasma. Notably, the fast ${\mathbf{v}}_{{\mathrm{E}} \times {\mathrm{B}}}^{RZ}$ moves not only lighter electrons but also heavier ions at the same speed in the RZ plane that is $v_{\mathrm{e}}^{ \bot ,RZ} \approx v_{\mathrm{i}}^{ \bot ,RZ} \approx v_{{\mathrm{E}} \times {\mathrm{B}}}^{RZ}$ (Figs. 3c and 6d and Supplementary Movie 4). When the plasma density exceeds a perpendicular critical density

3 $$n_{{\mathrm{crit}}, \bot } \equiv \left( {{\it{\epsilon }}_0B^2/m_{\mathrm{e}}} \right)\tan ^2\left( {\theta _B} \right){\mathrm{\gamma }}^{ - 2}$$

these newly enhanced ExB transports could become the most dominant transport mechanism in the RZ plane because of the very small *θ*_*B*_, yielding $v_{\mathrm{e}}^{ \bot ,RZ} \approx v_{\mathrm{i}}^{ \bot ,RZ} \ge v_{\mathrm{e}}^\parallel \sin \theta _B \gg v_{\mathrm{i}}^\parallel \sin \theta _B$ (Figs. 3c, 5c, and see Methods for details). The dominant ExB transports strongly influence the avalanche phenomena to differ completely from any electrical breakdown of the unmagnetized system. The roles of multi-scale ExB transports can be classified into two types that have distinct time and length scales: Faster turbulent ExB mixing and diffusion of the plasma along **B**_*RZ*_ by fine structure of fluctuating self-electric fields, and slower mean ExB flow across **B**_*RZ*_ by broad structure of $\langle \overline {{\mathbf{E}}_{{\mathrm{self}}}^{RZ}} \rangle$.

### Faster turbulent ExB mixing and diffusion along B_RZ_

**Fig. 7:**
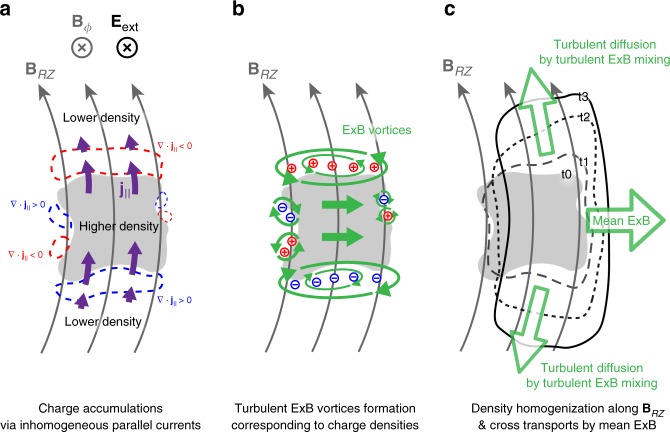
Turbulent diffusion along B_*RZ*_ by turbulent ExB mixing. **a** The space-charges (*ρ*) accumulate at the regions of high parallel density gradient ($\nabla _\parallel n_e$) due to the inhomogenous parallel plasma currents (purple): $\partial \rho /\partial {\mathrm{t}} = - \nabla \cdot {\mathbf{j}}_\parallel$. Positive and negative charges accumulate at the convergent ${\mathbf{j}}_\parallel$ region (red dashed line) and the divergent ${\mathbf{j}}_\parallel$ region (blue dashed line), respectively. **b** Turbulent ExB vortices induced by the space-charges mix the inhomogeneous plasma density along their trajectories to yield homogeneity. **c** Repetitive charge accumulation and ExB mixing cause a turbulent diffusion along **B**_*RZ*_ homogenizing the plasma density along **B**_*RZ*_. At the same time, the slower mean ExB moves the plasma across **B**_*RZ*_

The turbulent ExB mixing and the subsequent turbulent diffusion of the plasma are caused by the fluctuating local space-charge and corresponding self-electric field. It is worthwhile to address how the local space-charge accumulates in the plasma to understand the mechanism of the turbulent ExB mixing. The toroidal external electric fields induce the parallel plasma currents proportional to the plasma density that is **j**_||_ ~ *n*_e_*q*_e_**v**_de,||_ where **v**_de,||_ is the parallel electron drift velocity along the magnetic fields. The parallel plasma currents are inhomogeneous due to the inhomogeneous plasma density in the device. The local space-charge density (*ρ*) accumulates in the space as the convergence of the inhomogeneous parallel plasma currents, $\frac{{\partial \rho }}{{\partial t}} = - \nabla \cdot {\mathbf{j}}_\parallel$ (Fig. 7a). Since the divergence of the parallel plasma currents is mainly proportional to the parallel gradient of the plasma density ($\nabla _\parallel n_{\mathrm{e}}$), the stronger local space-charge accumulates at the higher parallel density gradient region ($\nabla _\parallel \cdot {\mathbf{j}}_\parallel \approx n_e{\mathbf{v}}_{de,\parallel } \cdot \nabla _\parallel n_e$). The resulting local space-charge produces an ExB vortex encompassing itself whose vorticity is determined by the local space-charge density, $\nabla \times {\mathbf{v}}_{{\mathrm{E}} \times {\mathrm{B}}} \approx - \rho /\left( {{\it{\epsilon }}_0B} \right){\hat{\mathbf b}}$ (Supplementary Movie 2). For example, Figs 3e, 5a, and 7b show that the divergent and convergent self-electric fields encompassing the charge density at the edge of the main plasma can produce ExB vortices in the clockwise and counter-clockwise directions, respectively. Notably, the ExB vortices are strongly turbulent because the viscosity of the plasma due to the Coulomb collisions is negligible during the breakdown. Also, the speed of the ExB vortices is much faster than the mean ExB flow since the fluctuating self-electric fields are much stronger than the mean self-electric fields $\langle \overline {{\mathbf{E}}_{{\mathrm{self}}}^{RZ}} \rangle$ (Fig. 4d, e). Therefore, the turbulent ExB vortices rapidly mix the plasma density along their trajectories connecting the low-density-plasma and the high-density-plasma. This turbulent ExB mixing actively homogenizes the plasma density along **B**_*RZ*_ until it sufficiently mitigates the parallel density gradient $\nabla _\parallel n_e$, because $\nabla _\parallel n_e$ and hence the space-charge accumulation are the free-energy-sources of the turbulent ExB vortices. Therefore, the continuous turbulent ExB mixing causes a turbulent plasma diffusion along **B**_*RZ*_ resulting in the homogenization of the parallel plasma density.

**Fig. 8:**
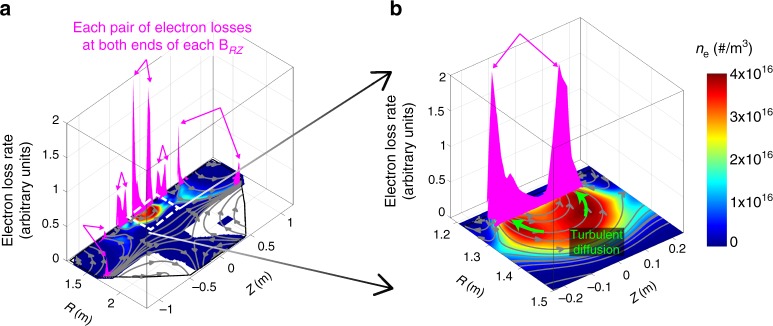
Dominant plasma losses due to the turbulent diffusion along **B**_*RZ*_. **a** Color contour plot indicates the linear-scale electron density in the RZ plane. The magnitude of the electron loss to the wall is represented in magenta along the wall trajectory. Each pair of magenta arrows indicates each pair of the electron losses at both ends of each poloidal magnetic field line (**B**_*RZ*_). **b** Enlarged graphs of the electron density and loss at the inboard center. The turbulent diffusion (green) along **B**_*RZ*_ by the turbulent ExB mixing causes the pair of electron losses with the same magnitude (see Supplementary Movie 3 for details)

The ExB mixing and the subsequent turbulent diffusion along **B**_*RZ*_ play crucial roles in the ohmic breakdown. First, the fast turbulent diffusion causes the homogeneous plasma density profile along **B**_*RZ*_ that is entirely different from the Townsend theory’s prediction of the exponential density profile (Fig. 6e, f, and Supplementary Movie 1). This turbulent ExB mixing mechanism successfully unravels the mystery of the homogeneous plasma structure along magnetic field lines observed in the experiments. Second, the turbulent diffusion along **B**_*RZ*_ dramatically enhances the plasma loss to the wall at both ends of each magnetic field line. Figure 8 shows that dominant plasma loss during the ohmic breakdown consists of several pairs of the plasma loss along each magnetic field line by the turbulent diffusion. Therefore, this enhanced plasma loss by the turbulent diffusion is another key mechanism responsible for the mysterious slow plasma formation of the ohmic breakdown (see Supplementary Note 1 how the ExB mixing makes a difference between the ohmic breakdown and the streamer).

### Slower mean ExB flow across B_RZ_

We now focus on a slower part of the ExB transport caused by the temporal averaged self-electric fields $\langle \overline {{\mathbf{E}}_{{\mathrm{self}}}^{RZ}} \rangle$, so-called the mean ExB. While the parallel component of $\langle \overline {{\mathbf{E}}_{{\mathrm{self}}}^{RZ}} \rangle$ cancels $E_{{\mathrm{ext}}}^{\phi ,\parallel }$, the perpendicular component of $\langle \overline {{\mathbf{E}}_{{\mathrm{self}}}^{RZ}} \rangle$ induces the mean ExB flow. The mean ExB flow $\langle \overline {{\mathbf{v}}_{{\mathrm{E}} \times {\mathrm{B}}}^{RZ}} \rangle$ moves the plasma across **B**_*RZ*_, and its maximum magnitude is $\left( {E_{{\mathrm{ext}}}^\phi /B_{RZ}} \right)\cos \theta _B$ as follows:

4 $$\langle \overline {{\mathbf{v}}_{{\mathrm{E}} \times\! {\mathrm{B}}}^{RZ}} \rangle= \frac{{\langle \overline {{\mathbf{E}}_{{\mathrm{self}}}^{RZ} } \rangle \times {\mathbf{B}}_\phi }}{{B^2}} = \frac{{\left| {{\mathbf{E}}_{{\mathrm{ext}}}^\phi } \right|}}{{\left| {{\mathbf{B}}_{RZ}} \right|}}\cos \theta _B\left( {{\hat{\mathbf E}}_{{\mathrm{ext}}}^\phi \times {\hat{\mathbf B}}_{RZ}} \right)$$

**Fig. 9:**
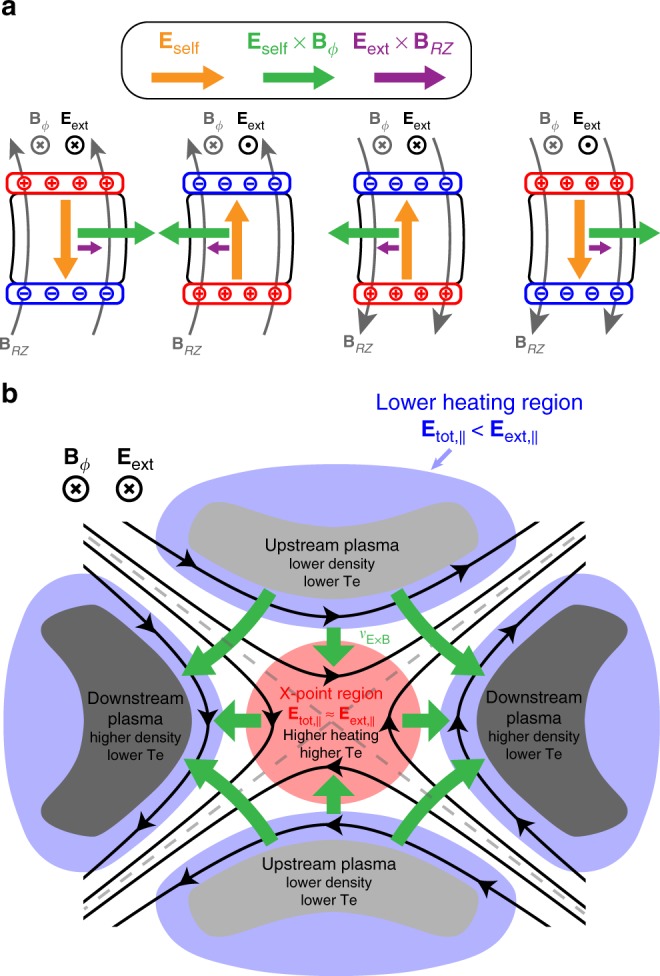
Comprehensive plasma dynamics in the complex EM topology. **a** Topological relationships between the external EM fields, corresponding space-charges, mean self-electric fields $\langle \overline {{\mathbf{E}}_{{\mathrm{self}}}^{RZ}} \rangle$ (orange), self-induced mean ExB drift in the direction of $\langle \overline{{\mathbf{E}}_{{\mathrm{self}}}^{RZ}} \rangle \times {\mathbf{B}}_\phi$ (green), and externally-driven ExB drift in the direction of ${\mathbf{E}}_{{\mathrm{ext}}}^\phi \times {\mathbf{B}}_{RZ}$ (purple) in various combinations of EM fields. **b** Topology analysis of the quadrupole EM structure regarding the X-point that determines overall structure and behaviors of the plasma evolution. The magnitude of the total parallel electric field in the X-point region (red) is larger than that in the other regions (blue). The mean ExB drifts (green) corresponding to the quadrupole magnetic structure yield two inflows and two outflows around the X-point. The plasma density of the downstream region (dark gray) is higher than that of the upstream region (pale gray)

Figure 9a clearly depicts relationships between the external EM fields, the mean self-electric fields, and the mean ExB flow. Interestingly, the direction of the mean ExB flow is highly predictable from the external EM fields (Fig. 9a), because it has the same direction of the cross product between the external toroidal electric field and the poloidal magnetic field as Eq. (4) describes. The mean ExB flow is the most dominant transport mechanism across **B**_*RZ*_ because it is several orders of magnitudes higher than the characteristic speed of the collisional diffusion across **B**_*RZ*_. Therefore, the plasma moves across **B**_*RZ*_ by the mean ExB during the ohmic breakdown, while the plasma density is homogeneous along each **B**_*RZ*_ due to the turbulent ExB mixing and diffusion. The mean ExB flow plays an essential role to understand the overall movements of the plasma in the complex electromagnetic structure. Notably, the position of the main plasma (highest-density) at the end of the ohmic breakdown phase could be easily predicted by analyzing the directions of the mean ExB flows in the given electromagnetic structure.

### Comprehensive plasma dynamics in the complex EM topology

The understanding of the parallel and perpendicular plasma responses to the external EM fields led us to discover a comprehensive plasma dynamics in the complex EM topology especially for a quadrupole structure of the poloidal magnetic fields around the X-point where the magnitude of the poloidal field is zero (|**B**_*RZ*_| = 0). The comprehensive plasma dynamics by considering the self-electric fields differ significantly from previous reports in the literature. For example, earlier studies on the location of the plasma formation assumed that the charged particles stay in the X-point region for a long time until they eventually escaped the very long magnetic field lines in the X-point region via the parallel transport. However, we found that the charged particles easily leave the X-point region because the plasma can rapidly move to the other region by the dominant ExB transports induced by the self-electric fields. Therefore, topological analysis of the plasma dynamics by considering the self-electric fields is essential to understand the mechanisms of the plasma formation in the complex electromagnetic structure.

The topological analysis of the plasma dynamics consists of the parallel and the perpendicular plasma responses to the quadrupole magnetic structures. The critical parallel density (*n*_crit,||_) is the criterion for determining the parallel plasma response whether the self-electric field of the plasma can cancel the parallel component of the external electric field ($E_{{\mathrm{ext}}}^{\phi ,\parallel }$) so that the ohmic heating power decreases. Notably, the closer to the X-point, the smaller magnetic pitch angle ($\theta _B^{{\mathrm{X}} - {\mathrm{point}}} \to 0$) causes infinitely large critical parallel density towards the X-point ($n_{{\mathrm{crit}},\parallel }^{{\mathrm{X}} - {\mathrm{point}}} \propto \cot ^2\left( {\theta _B^{{\mathrm{X}} - {\mathrm{point}}}} \right) \to \infty$) (Supplementary Fig. 5a). This diverging *n*_crit,||_ towards the X-point implies that a plasma of a certain density *n* cannot cancel $E_{{\mathrm{ext}}}^{\phi ,\parallel }$ at the X-point region due to the infinitely large $n_{{\mathrm{crit}},\parallel }^{{\mathrm{X}} - {\mathrm{point}}}$ ($n \ll n_{{\mathrm{crit}},\parallel }^{{\mathrm{X}} - {\mathrm{point}}} \approx \infty$) whereas it can cancel $E_{{\mathrm{ext}}}^{\phi ,\parallel }$ at other regions relatively far from the X-point due to a much lower $n_{{\mathrm{crit}},\parallel }^{{\mathrm{other}}}$ there ($n\, > \,n_{{\mathrm{crit}},\parallel }^{{\mathrm{other}}}$). Therefore, the X-point region has a larger total electric field ($E_{{\mathrm{tot}}}^\parallel \approx E_{{\mathrm{ext}}}^{\phi ,\parallel }$) and a higher ohmic heating power so consequently a higher plasma temperature. On the other hand, the other regions have reduced total electric fields ($\left| {E_{{\mathrm{tot}}}^\parallel } \right| = \left| {{E}_{{\mathrm{ext}}}^{\phi ,\parallel } + E_{\mathrm{self}}^{RZ,\parallel }} \right| < \left| {{E}_{{\mathrm{ext}}}^{\phi ,\parallel }} \right|$) and lower ohmic heating powers so consequently lower plasma temperatures. Interestingly, in contrast to *n*_crit,||_, the critical perpendicular density ($n_{{\mathrm{crit}}, \bot }$) becomes smaller towards the X-point ($n_{{\mathrm{crit}}, \bot }^{{\mathrm{X}} - {\mathrm{point}}} \propto \tan ^2\left( {\theta _B^{{\mathrm{X}} - {\mathrm{point}}}} \right) \to 0$) (Supplementary Fig. 5b). This infinitesimal $n_{{\mathrm{crit}}, \bot }^{{\mathrm{X}} - {\mathrm{point}}}$ implies that the plasma of the certain density *n* in the X-point region can easily produce dominant ExB transports overwhelming the parallel transports ($n \gg n_{{\mathrm{crit}}, \bot }^{{\mathrm{X}} - {\mathrm{point}}} \approx 0$). The turbulent ExB actively mixes the plasma density near the X-point, and in average, there are two mean ExB inflows and two mean ExB outflows around the X-point due to the quadrupole structure of the poloidal magnetic fields (Fig. 9b).

Overall, the topological analysis of plasma dynamics in the complex EM structure including the X-point can be distinguished into three stages. First, the colder plasmas with the lower growth rate in the upstream regions move into the X-point region by the mean ExB inflows. Second, the X-point region efficiently heats the plasma by the higher ohmic heating power so that the plasma temperature and the growth rate increase. Third, the heated plasma moves out to the downstream regions by the mean ExB outflows, and the plasma temperature and the growth rate decrease again due to the lower ohmic heating power at the downstream regions. Since the plasma density continually increases during these processes, the plasma density at the downstream region becomes higher than that of the upstream and X-point region. (Fig. 9b). Therefore, the main plasma of high-density locates at the downstream regions rather than the X-point region. This topology analysis regarding the X-point is significantly different from the previous understanding and methodologies that predict the main plasma would be produced at the X-point region.

**Fig. 10:**
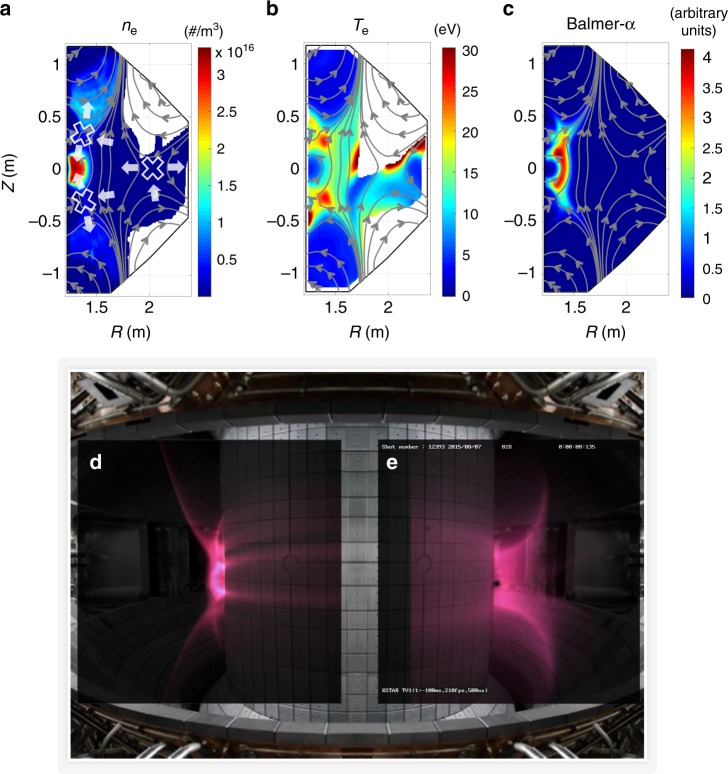
Comparison between BREAK simulation results and KSTAR experiments. Simulation results of **a** electron density, **b** electron temperature, and **c** Balmer-α line emissions at *t* = 36 ms of the KSTAR reference breakdown scenario. Bold white X symbols and white arrows in **a** indicate the locations of X-points and the directions of the mean ExB flows, respectively. **d** A synthetic diagnostic of the Balmer-α line emission in the simulation is compared with **e** an experimental visible camera image from KSTAR

The BREAK simulation clearly demonstrates the plasma dynamics regarding the inhomogeneous plasma density and temperature around the X-points (Fig. 10 and Supplementary Figs 6, 10d, e). For example, the KSTAR ohmic breakdown scenario has a complex electromagnetic structure with several X-points (Fig. 10a). The electron temperature is higher at each X-point region and along connected magnetic field lines (Fig. 10b and Supplementary Fig 6b), because the plasmas are efficiently heated at each X-point region and diffuse rapidly along **B**_*RZ*_ by the turbulent ExB mixing. At the same time, the mean ExB flows across **B**_*RZ*_ move the plasmas from the upstream to the downstream of each X-point in the device. As a result, the electron density is highest at the central inboard side where the final downstream region of the several X-points (Fig. 10a Supplementary Movie 4, 5). A Balmer-α emission structure (Fig. 10c) could differ from the plasma density structure (Fig. 10a) due to the strongly inhomogeneous electron temperature (Fig. 10b). The Balmer-α emission structure is mainly observed at the X-point regions and along connected magnetic field lines, because the electrons in the higher plasma temperature region can only exceed the threshold energy of the Balmer-α emission reaction. Notably, a synthetic diagnostic of the Balmer-α emission in the BREAK simulation reproduces the experimental Balmer-α emission structure at the inboard side along the magnetic field lines of the KSTAR device (Fig. 10c–e).The differences in the detailed structure of the emission could result from unavoidable uncertainties in the initial conditions of the simulation, magnetic field structure, and synthetic diagnostics.

## Discussion

We can consider two types of the plasma responses in general plasma physics: the self-electric field produced by the space-charge and the self-magnetic field induced by the plasma currents. In this study, we focused on the critical roles of the self-electric fields during the ohmic breakdown rather than the self-magnetic fields, although BREAK simulation systematically considers the poloidal self-magnetic fields produced by the toroidal plasma currents. This is because the self-magnetic fields play a little role during the ohmic breakdown phase. The small plasma current during the ohmic breakdown cannot produce considerable self-magnetic fields to change the plasma dynamics or the total magnetic structure significantly. We found that the small self-magnetic fields can only slightly change the positions of the X-points without a significant modification of the overall magnetic structure (Supplementary Fig. 7). The plasma density and currents should be much higher than that of the ohmic breakdown phase to change the overall magnetic structure such as the formation of the closed magnetic surface. For example, in the KSTAR experiments, the formation of the closed magnetic surface requires large plasma currents of several tens of kA with the high plasma density over 10^18^ m^−3^. On the other hand, even small plasma density about 10^12^ m^−3^ to 10^14^ m^−3^ can produce considerable self-electric fields that significantly changes the plasma dynamics. Therefore, the self-electric field rather than the self-magnetic field dominantly influences the ohmic breakdown physics.

The ExB transports in the ohmic breakdown phase are distinct from other ExB transports in the tokamak discharges. It is worthwhile to compare the transport mechanism of the ohmic breakdown plasma and that of the SOL (Scrape Off Layer) plasma^27^ because both cases have open magnetic field lines but different magnetic pitch angles and different space-charge separation mechanisms. The magnetic pitch angle of the ohmic breakdown (*θ*_*B*_ ≤ 10^−3^) is 2–3 orders of magnitude smaller than that of the usual SOL region (*θ*_*B*_ ~ 10^−1^). In the ohmic breakdown plasma, the toroidal electric field directly separates the space-charge along **B**_*RZ*_. On the contrary, in the conventional SOL plasma, the space-charge slowly separates in the Z-direction by the ∇*B* and curvature drift. The very small magnetic pitch angle of the ohmic breakdown (*θ*_*B*_ ≤ 10^−3^) significantly reduces the magnitude of the parallel transports in the RZ plane ($v_{\mathrm{e}}^{\parallel ,RZ} = v_{\mathrm{e}}^\parallel \sin \theta _B \ll v_{\mathrm{e}}^\parallel$ and $C_{\mathrm{s}}^{\parallel ,RZ} = C_{\mathrm{s}}^\parallel \sin \theta _B \ll C_{\mathrm{s}}^\parallel$), so that the ExB transports could overwhelm the parallel transports ($\left| {{\mathbf{v}}_{{\mathrm{E}} \times {\mathrm{B}}}} \right| > v_{\mathrm{e}}^{\mathrm{th}}\sin \theta _B \gg C_{\mathrm{s}}\sin \theta _B$). Therefore, in the ohmic breakdown, the turbulent ExB mixing rapidly diffuses the plasma along **B**_*RZ*_ while the mean ExB moves the plasma across **B**_*RZ*_ in the direction of ${\hat{\mathbf E}}_{{\mathrm{ext}}}^\phi \times {\hat{\mathbf B}}_{RZ}$ that can has an arbitrary direction depending on the situation (Eq. 4, Fig. 9). On the contrary, in the SOL plasma, the ion sound speed $C_{\mathrm{s}}^{\parallel ,RZ}$ is the dominant transport mechanism along **B**_*RZ*_ due to the considerable magnetic pitch angle (*θ*_*B*_~10^−1^), and the ExB moves the plasma across **B**_*RZ*_ that is usually outward radial direction due to the charge separation in the Z-direction.

The topology analysis of external EM fields regarding the X-point (Fig. 9b and Supplementary Fig. 5) provides a successful prediction of the overall structure and behavior of the plasma evolution. This systematic prediction significantly influences the design strategy of breakdown scenarios (Supplementary Figs. 8, 9). Previously, the main plasma location and its behavior during breakdown were poorly understood and therefore not carefully considered in the scenario design. The field-null (X-point) region was considered to be the location of the main plasma and the magnitudes of external EM fields have been considered as a primary design parameter such as the empirical condition (Supplementary Fig. 9a, b). However, we can deduce the actual plasma evolution based on the magnitudes and the directions of the EM fields by considering the self-electric fields and related transports. Notably, the X-point locations and their topological relations determine the overall structure and behavior of the plasma evolution. One can easily predict the main plasma position from the topological relationship between X-points (Supplementary Fig. 9c, d) before conducting expensive simulations (Supplementary Fig. 10d, e, and Supplementary Movie 5) or experiments, because the plasma will exhibit higher density in the downstream region of each X-point. Therefore, it is now possible to determine the plasma position and behavior during the design stage by using the topology analysis of the external EM fields (Fig. 9b and Supplementary Fig. 9c, d). This topology analysis as a new design strategy facilitates the design of robust and optimized breakdown scenarios for currently existing and future tokamaks, such as ITER and beyond.

## Methods

### Plasma growth rate analysis on KSTAR breakdown experiments

We analyzed the plasma growth rates for about 948 shots of KSTAR ohmic breakdown experiments to verify whether the Townsend theory is valid or not. Since the diagnostics measruing the plasma density in KSTAR are designed for hot and dense fusion plasmas, it is difficult to directly measure the growth rate of the low-density-plasma during the ohmic breakdown. Instead, the growth rate of the electron density (*n*_e_) can be calculated from the growth rate of the plasma current (*I*_p_) by the electron avalanche that is $\frac{1}{{n_{\mathrm{e}}}}\frac{{\mathrm{d}\it{n}_{\mathrm{e}}}}{{\mathrm{d}\it{t}}} \approx \frac{1}{{I_{\mathrm{p}}}}\frac{{\mathrm{d}\it{I}_{\mathrm{p}}}}{{\mathrm{d}\it{t}}}$. This equivalent relation between the relative growth rate of the plasma density and current is valid in the ohmic breakdown phase because the growth rate of the electron drift velocity (*v*_d,e_) is negligible than the growth rate of the plasma density ($\frac{{{\mathrm{d}}v_{{\mathrm{d}},{\mathrm{e}}}}}{{\mathrm{d}\it{t}}} \ll \frac{{\mathrm{d}\it{n}_{\mathrm{e}}}}{{\mathrm{d}\it{t}}}$) as follows:

$$\frac{1}{{I_{\mathrm{p}}}}\frac{{\mathrm{d}\it{I}_{\mathrm{p}}}}{{\mathrm{d}\it{t}}} = \frac{1}{{n_{\mathrm{e}}q_{\mathrm{e}}v_{{\mathrm{d}},{\mathrm{e}}}}}\frac{{\mathrm{d}\left( {\it{n}_{\mathrm{e}}q_{\mathrm{e}}v_{{\mathrm{d}},{\mathrm{e}}}} \right)}}{{\mathrm{d}\it{t}}} \approx \frac{1}{{n_{\mathrm{e}}}}\frac{{\mathrm{d}\it{n}_{\mathrm{e}}}}{{\mathrm{d}\it{t}}}$$

where *q*_e_ is the electron charge. The plasma current is a good alternative to analyze the plasma growth rate because the plasma current is much more reliable experimental data than the plasma density at the start-up phase. The Rogowski coils can measure the total plasma currents integrated over the RZ plane, while the interferometer only measures the line-integrated plasma density at few local points. Therefore, the growth rate of the total plasma current well represents the growth rate of the total plasma density in the device. We can estimate the relative current growth rate ($\frac{1}{{I_{\mathrm{p}}}}\frac{{\mathrm{d}\it{I}_{\mathrm{p}}}}{{\mathrm{d}\it{t}}}$) from the experiments and compare them with the Townsend theory’s prediction for the relative density growth rate ($\frac{1}{{n_{\mathrm{e}}}}\frac{{\mathrm{d}\it{n}_{\mathrm{e}}}}{{\mathrm{d}\it{t}}}$). The relative density growth rate predicted by the Townsend theory can be calculated from following formulas^10^:

$$\begin{array}{*{20}{l}} {\frac{1}{{n_{\mathrm{e}}}}\frac{{\mathrm{d}n_{\mathrm{e}}}}{{\mathrm{d}t}}} \hfill & = \hfill & {v_{{\mathrm{d}},{\mathrm{e}}}\left( {\alpha - \frac{1}{{L_{{\mathrm{eff}}}}}} \right)} \hfill \\ \alpha \hfill & = \hfill & {510{\kern 1pt} p{\kern 1pt} {\mathrm{exp}}\left( { - \frac{{1.25 \times 10^4p}}{E}} \right)} \hfill \\ {v_{{\mathrm{d}},{\mathrm{e}}}} \hfill & = \hfill & {43\left( {E{\mathrm{/}}p} \right)} \hfill \end{array},$$

where *α* is the first Townsend coefficient, *v*_d,e_ is the is the electron drift velocity, *L*_eff_ is the effective connection length of the magnetic fields (Supplementary Fig. 1c, d), *p* is the gas pressure in Torr unit, and *E* is the magnitude of the electric field in V m^−1^ unit. The three parameters of *E*, *p*, and *L*_eff_ need to be carefully determined from the experimental data because of the uncertainties of the diagnostics and the plasma position. As a statistical analysis, we scan the allowable ranges of each parameter to overcome the uncertainties. The plasma position is in the range of R = 1.2–2.4 m, *L*_eff_ is in the range of 500–1500 m, and the gas pressure at the plasma is in the range of 1-3 times the pressure at the vacuum pump. This statistical analysis gives the average and error bars of the relative growth rate for each shot so that we can avoid excessively overestimated or underestimated values due to the uncertainties. Figure 2 shows the comparison between the experimental data and the Townsend theory’s predictions regarding the relatvie growth rate. This clearly indicates that the experimental relative growth rate of the plasma is 1-2 orders of magnitude slower than that of the Townsend theory’s prediction.

### BREAK code

BREAK^23^ is an electrostatic particle simulation code for the cylindrical coordinate system to study the ohmic breakdown phenomena in a tokamak, self-consistently. The BREAK code is written in the C/C++ language. Since the ohmic breakdown phenomena span a broad range of spatial-temporal scales, such as the fast electron gyro-motion in picoseconds and the slow plasma evolution in milliseconds, various physical and numerical models are adopted to simulate desirable breakdown phenomena of our interest within a reasonable computation time.

The particle-in-cell (PIC) method^28^ is adopted to deal with collective behaviors of the plasma in the cylindrical coordinate system. There are two choices for the dimension of the problem in the BREAK code: 2D (R–Z) coordinates by assuming the toroidally symmetric plasma or full 3D (R-*ϕ*-Z) coordinates without any symmetric assumptions. An equation of adiabatic charged particle motion^29^ is discretized by using a D1 damping scheme^30,31^ to calculate the guiding center motion of the charged particles without dealing with uninteresting fast gyromotions. The gyrokinetic Poisson equation^32^ is adopted to calculate the self-electric fields produced by the plasma space-charges with considering the polarization shielding effects via the ion polarization drifts in the Debye length scales. The equation of charged particle motion and the gyrokinetic Poisson equation are coupled by using direct-implicit methods^33–39^ to overcome the temporal restriction coming from the plasma oscillation, ${\mathrm{\Delta t}} < {\mathrm{\omega }}_{{\mathrm{pe}}}^{ - 1}$, where Δt is the time step size and ω_pe_ is the plasma frequency.

Six species, e^−^, ${\mathrm{H}}_2^ +$, ${\mathrm{H}}_3^ +$, H^+^, H^0^, and ${\mathrm{H}}_2^0$, and three types of collisional processes are considered in the BREAK code. The total 26 impact collision reactions between the simulation particles and the background hydrogen neutral gas are dealt with the MonteCarlo collision (MCC) method^40^. The MCC method is also applied to plasma–wall interactions such as the secondary electron emissions. Coulomb collisions between the charged particles are calculated by Nanbu’s algorithms^41,42^. Since the plasma density and corresponding numerical particles increase exponentially during the electron avalanche, particle coalescence strategies, such as modified binary scheme^43^ and adaptive particle management (APM)^44^, are introduced to control the number of numerical particles while preserving the plasma density, temperature, and momentum. The poloidal self-magnetic fields ${\mathbf{B}}_{{\mathrm{self}}}^{RZ}$ by the two-dimensional toroidal plasma currents *J*_*ϕ*_(*R*, *Z*) are calculated by solving the Ampere’s law $\nabla \times {\mathbf{B}}_{{\mathrm{self}}}^{RZ} = \mu _0J_\phi \hat \phi$. The self-magnetic fields ${\mathbf{B}}_{{\mathrm{self}}}^{RZ}$ are added to the external poloidal magnetic fields ${\mathbf{B}}_{{\mathrm{ext}}}^{RZ}$ to obtain the total magnetic fields ${\mathbf{B}}_{{\mathrm{tot}}}^{RZ}$ (${\mathbf{B}}_{{\mathrm{tot}}}^{RZ} = {\mathbf{B}}_{{\mathrm{ext}}}^{RZ} + {\mathbf{B}}_{{\mathrm{self}}}^{RZ}$), so that BREAK systematically considers the modification of the poloidal magnetic field structure due to the toroidal plasma currents. The BREAK code is parallelized by adopting MPI^45^ and OpenMP^46^ frameworks to reduce the total computation time.

### Simulations of the KSTAR reference breakdown scenario

BREAK is applied to the reference ohmic breakdown scenario of the KSTAR tokamak to investigate the dynamic plasma evolution in a realistic situation. Supplementary Fig. 2 shows the experimental results of the reference ohmic breakdown scenario. The time-varying external electromagnetic structures during the breakdown are calculated by using a non-linear model that considers eddy currents in the wall segments and ferromagnetic incoloy 908 material effects^26^ (Supplementary Fig. 1). The static toroidal magnetic fields of $B_\phi = \left( { - 2.7 \times 1.8} \right)R^{ - 1}\hat \phi$ are applied in the device. As initial conditions, the pre-filled gas pressure is 4 mPa equivalent to the H_2_ molecule density of 8.3 × 10^17^ m^−3^ at the room temperature of 0.03 eV. The initial plasma density and the temperature are assumed to be 10^6^ m^−3^ and the room temperature of 0.03 eV, respectively, which are uniformly distributed in the device. Notably, the physical mechanisms and the general trend of the electron avalanche in the tokamak does not rely on the magnitude of the initial plasma density. The different initial plasma densities result in small time shifts of the plasma density and current at the end of ohmic breakdown phase, which agree with the experimental observations. We simulated two different cases, with and without considering self-electric fields, for the same initial and boundary conditions to study the roles of the self-electric fields during the ohmic breakdown. Figure 6 presents the particle simulation results for these two cases (Supplementary Movie 1). Figure 10 shows comparison of the simulation results and the KSTAR experiment. More detailed simulation results are shown in Supplementary Fig. 6 and Supplementary Movie 1–4.

### Artificial single X-point scenarios

Artificial single X-point scenarios are designed to investigate the basic ohmic breakdown physics in a simplified situation. External EM fields in these scenarios are static over time. Toroidal magnetic fields of $B_\phi = \left( { - 1.5 \times 2.0} \right)R^{ - 1}\hat \phi$ and uniform loop voltage of 10 V are applied. Vertical magnetic fields **B**_*RZ*_ are designed to have a single X-point (|**B**_*RZ*_ | = 0) at the center of the device. To identify the role of directions of the EM fields, we considered two different cases; forward and reversed **B**_*RZ*_, that have the same magnitude but opposite direction of the vertical magnetic fields as shown in Supplementary Fig. 8. These two cases have exactly the same magnitudes of EM fields. Supplementary Fig. 9 shows the differences between the traditional analysis of external field quality (empirical condition) and the topology analysis method proposed in this paper for the two cases. Particle simulation results (Supplementary Fig. 10 and Supplementary Movie 5) clearly demonstrate that the plasma position and the behavior strongly depend on the direction of the EM fields and agree well with the prediction by the topology analysis method.

### Derivation of critical densities

We derived the critical densities, *n*_crit,||_ and $n_{{\mathrm{crit}}, \bot }$, for the symmetric plasma and uniform electromagnetic fields in the Cartesian coordinate system for simplicity. The plasma shape is a rectangle of width w and length L in the vertical (*x*–*z*) plane. The characteristic length of the charge separation at the plasma edge is the Debye length $\lambda _{\mathrm{D}} = \sqrt {{\it{\epsilon }}_0kT_e/ne^2}$ where *ε*_0_ is the permittivity, *k* is the Boltzmann constant, *T*_e_ is the electron temperature, *n* is the plasma density, and *e* is the elementary charge. The characteristic strength of the self-electric field $E_{{\mathrm{self}}}^Z$ is calculated at the center of the plasma as following:

$$\begin{array}{*{20}{l}} {E_{{\mathrm{self}}}^Z} \hfill & = \hfill & {2\mathop {\displaystyle\int }\nolimits_{\hskip-5pt\left( {L - \lambda _{\mathrm{D}}} \right)/2}^{\left( {L + \lambda _{\mathrm{D}}} \right)/2} \mathop {\displaystyle\int }\nolimits_{\hskip-5pt - \infty }^\infty \mathop {\displaystyle\int }\limits_{\hskip-3pt - w/2}^{w/2} \frac{{ne}}{{4{\mathrm{\pi }}{\it{\epsilon }}_0}}\frac{{z_{\mathrm{s}}}}{{\left( {x_{\mathrm{s}}^2 \,+\, y_{\mathrm{s}}^2 \,+\, z_{\mathrm{s}}^2} \right)^{\frac{3}{2}}}}\mathrm{d}\it{x}_{\mathrm{s}}\mathrm{d}\it{y}_{\mathrm{s}}\mathrm{d}\it{z}_{\mathrm{s}}} \hfill \\ {} \hfill & = \hfill & {\frac{{ne}}{{2{\mathrm{\pi }}{\it{\epsilon }}_0}}\left[ {2\left( {L + \lambda _{\mathrm{D}}} \right){\mathrm{tan}}^{ - 1}\left( {\frac{w}{{L \,+\, \lambda _{\mathrm{D}}}}} \right) - 2\left( {L - \lambda _{\mathrm{D}}} \right){\mathrm{tan}}^{ - 1}\left( {\frac{w}{{L \,-\, \lambda _{\mathrm{D}}}}} \right)} \right.} \hfill \\ {} \hfill & {} \hfill & {\left. { + w{\kern 1pt} {\mathrm{ln}}{\kern 1pt} \left( {1 + \frac{{4L\lambda _{\mathrm{D}}}}{{w^2 \,+\, \left( {L \,-\, \lambda _{\mathrm{D}}} \right)^2}}} \right)} \right]} \hfill \\ {} \hfill & \approx \hfill & {\frac{{ne\lambda _{\mathrm{D}}}}{{{\it{\epsilon }}_0}}\left[ {\frac{2}{\pi }\tan ^{ - 1}\left( {\frac{w}{L}} \right)} \right]\;{\mathrm{if}}\,\lambda _{\mathrm{D}} \ll L} \hfill \\ {} \hfill & = \hfill & {\sqrt {\frac{{nkT_{\mathrm{e}}}}{{{\it{\epsilon }}_0}}} \gamma } \hfill \end{array}$$

where $\gamma \equiv \left( {2/{\mathrm{\pi }}} \right)\tan^{ - 1}\left( {w/L} \right)$ is a simplified shape factor by assuming small *λ*_D_/*L*. The shape factor *γ* becomes 1 for a very wide plasma (*w* >> *L*), and it becomes 0 for a very narrow plasma (*w* << *L*).

The parallel critical density *n*_crit,||_ is derived from a condition that the parallel component of the self-electric field ($E_{{\mathrm{self}}}^{Z,\parallel } = E_{{\mathrm{self}}}^Z\sin \theta _B$) is larger than the parallel external field ($E_{{\mathrm{ext}}}^{\phi ,\parallel } = E_{{\mathrm{ext}}}^\phi \cos \theta _B$) as follows:

$$\begin{array}{*{20}{l}} {E_{{\mathrm{self}}}^{Z,\parallel }} \hfill & \ge \hfill & {E_{{\mathrm{ext}}}^{\phi ,\parallel }} \hfill \\ {\sqrt {\frac{{nkT_{\mathrm{e}}}}{{{\it{\epsilon }}_0}}} \gamma {\kern 1pt} {\mathrm{sin}}{\kern 1pt} \theta _B} \hfill & \ge \hfill & {E_{{\mathrm{ext}}}^\phi {\kern 1pt} {\mathrm{cos}}{\kern 1pt} \theta _B} \hfill \\ {n} \hfill & \ge \hfill & {\left( {\frac{{{\it{\epsilon }}_0}}{{kT_e}}} \right){\kern 1pt} {\mathrm{cot}}^2{\kern 1pt} \theta _B\left( {E_{{\mathrm{ext}}}^\phi } \right)^2\gamma ^{ - 2} \equiv n_{{\mathrm{crit}},\parallel }} \hfill \end{array}$$

When the plasma density exceeds *n*_crit,||_, the plasma is capable of canceling the external electric fields as the plasma response, which is caused by a short charge separation within the Debye length scale.

The perpendicular critical density $n_{{\mathrm{crit}}, \bot }$ is derived from a condition that the perpendicular ExB drift in the RZ plane, $v_{{\mathrm{E}} \times {\mathrm{B}}}^{RZ} = E_{\mathrm{self}}^ZB_\phi /B^2 = E_{\mathrm{self}}^Z\cos \theta _B/B$, is faster than the parallel component of the electron thermal motion in the RZ plane, ${\mathrm{v}}_{{\mathrm{th}},{\mathrm{e}}}^{RZ} = v_{{\mathrm{th}},{\mathrm{e}}}\sin \theta _B$, as follows:

$$\begin{array}{*{20}{l}} {v_{{\mathrm{E}} \times {\mathrm{B}}}^{RZ}} \hfill & \ge \hfill & {v_{{\mathrm{th}},{\mathrm{e}}}^{RZ}} \hfill \\ {\sqrt {\frac{{nkT_{\mathrm{e}}}}{{{\it{\epsilon }}_0}}} \gamma \frac{{\cos \theta _B}}{B}} \hfill & \ge \hfill & {\sqrt {\frac{{kT_{\mathrm{e}}}}{{m_{\mathrm{e}}}}} {\kern 1pt} {\mathrm{sin}}{\kern 1pt} \theta _B} \hfill \\ {n} \hfill & \ge \hfill & {\left( {\frac{{{\it{\epsilon }}_0B^2}}{{m_{\mathrm{e}}}}} \right){\kern 1pt} {\mathrm{tan}}^2{\kern 1pt} \left( {\theta _B} \right)\gamma ^{ - 2} \equiv n_{{\mathrm{crit}}, \bot }} \hfill \end{array}$$

When the plasma density exceeds $n_{{\mathrm{crit}}, \bot }$, the perpendicular ExB transport is dominant over the parallel electron transport.

## Electronic supplementary material

Supplementary Information

Description of Additional Supplementary Files

Supplementary Movie 1

Supplementary Movie 2

Supplementary Movie 3

Supplementary Movie 4

Supplementary Movie 5

## Data Availability

The BREAK code belongs to Seoul National University. The code and data that support the findings of this study are available from the corresponding author upon reasonable request.
