# Supplementary material for: Evidence of a turbulent ExB mixing avalanche mechanism of gas breakdown in strongly magnetized systems
Source: Nat Commun. 2018 Aug 30;9:3523. doi: 10.1038/s41467-018-05839-5 (PMC6117305; doi:10.1038/s41467-018-05839-5)
Supplement: Supplementary file 1 — Supplementary Information [file 41467_2018_5839_MOESM1_ESM.pdf]

Evidence of a turbulent ExB mixing avalanche mechanism of gas breakdown in strongly magnetized systems

Min-Gu Yoo et al.

Supplementary Note 1

Supplementary Figures 1-10

Supplementary Note1. Comparison with the streamer theory

It is worthwhile to compare our novel mechanisms of the ohmic breakdown to the traditional streamer theory to understand the importance of the ExB transports. The electron avalanche of the ohmic breakdown in the RZ plane is similar to that of the streamer breakdown in terms of the high amplification of the single avalanche. Although the parallel plasma dynamics of the ohmic breakdown is similar to that of the streamer theory, the perpendicular dynamics make the ohmic breakdown differs from the streamer theory completely. For example, above the critical plasma density, the ions at the avalanche tail could produce considerable self-electric fields (Supplementary Figure 4b, c). The self-electric fields decrease the total parallel electric fields at the main plasma region, whereas they enhance the total fields at the avalanche tail region. In the case of the positive streamer, the seed electrons at the tail produced by the photoionization rapidly obtain high energy by the enhanced total electric fields. The accelerated seed electrons produce secondary avalanches towards the tail of the first avalanche so that the overall avalanche process dramatically accelerates (Supplementary Figure 4b). On the contrary, the avalanche process within the strongly magnetized system could be significantly delayed because of the turbulent ExB mixing. The seed electrons at the tail try to obtain the high energy by the enhanced total parallel electric fields. However, the turbulent ExB vortices at the avalanche tail try to mix and diffuse the seed electrons into other regions before they get enough energy to make the secondary avalanches. If the ExB transport is dominant over the parallel electron transports ($n > n_{\mathrm{crit},\perp}$), the turbulent ExB mixing prevents the secondary avalanches and diffuses the plasma along the magnetic field lines with the slow growth rate as described in the main manuscript.

Supplementary Figure 1

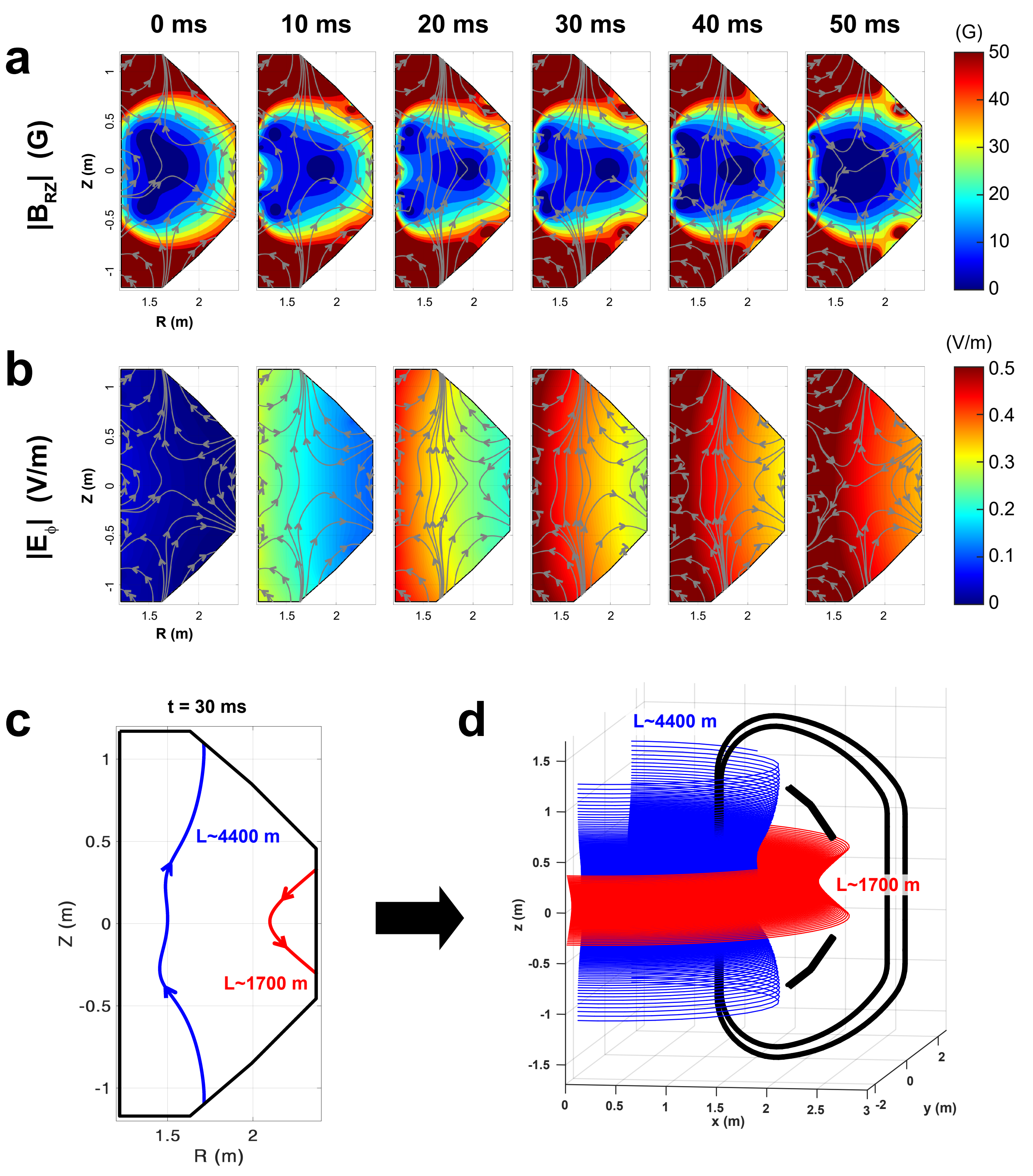

Supplementary Figure 1 | Externally driven electromagnetic structures in KSTAR. In the process of producing the toroidal electric fields, time-varying external electromagnetic structures are produced in the device. **a**, Vertical magnetic fields $\mathbf{B}_{RZ}$ and **b**, external toroidal electric fields have complex structures and inhomogeneous magnitudes in the RZ plane. Two sample magnetic field lines at t = 30 ms are depicted in **c**, RZ plane and **d**, 3-dimensional space. The length of each field line is 4400 m (blue) and 1700 m (red), respectively.

Supplementary Figure 2

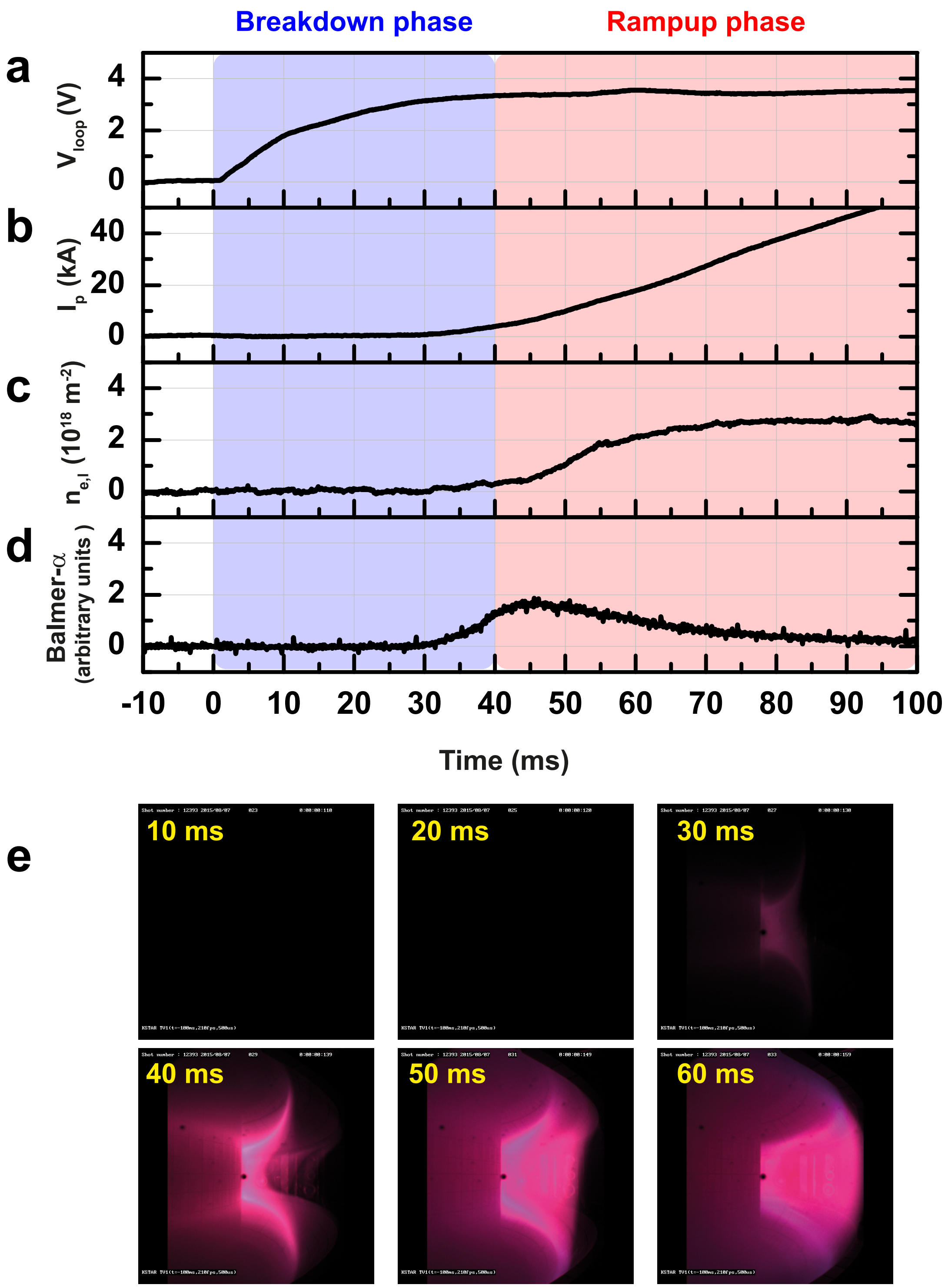

Supplementary Figure 2 | Experimental results of plasma start-up in KSTAR.
Temporal evolutions of **a**, applied loop voltage induced by central solenoids, **b**, plasma current, **c**, line-averaged plasma density, and **d**, line-integrated Balmer-α emission can be classified into two phases; ohmic breakdown of pre-filled neutral gas molecules (blue shaded box) and ramp-up of the plasma current (red shaded box). **e**, Snapshots of visible camera images during the plasma start-up that show homogeneous structures along the magnetic field lines.

Supplementary Figure 3

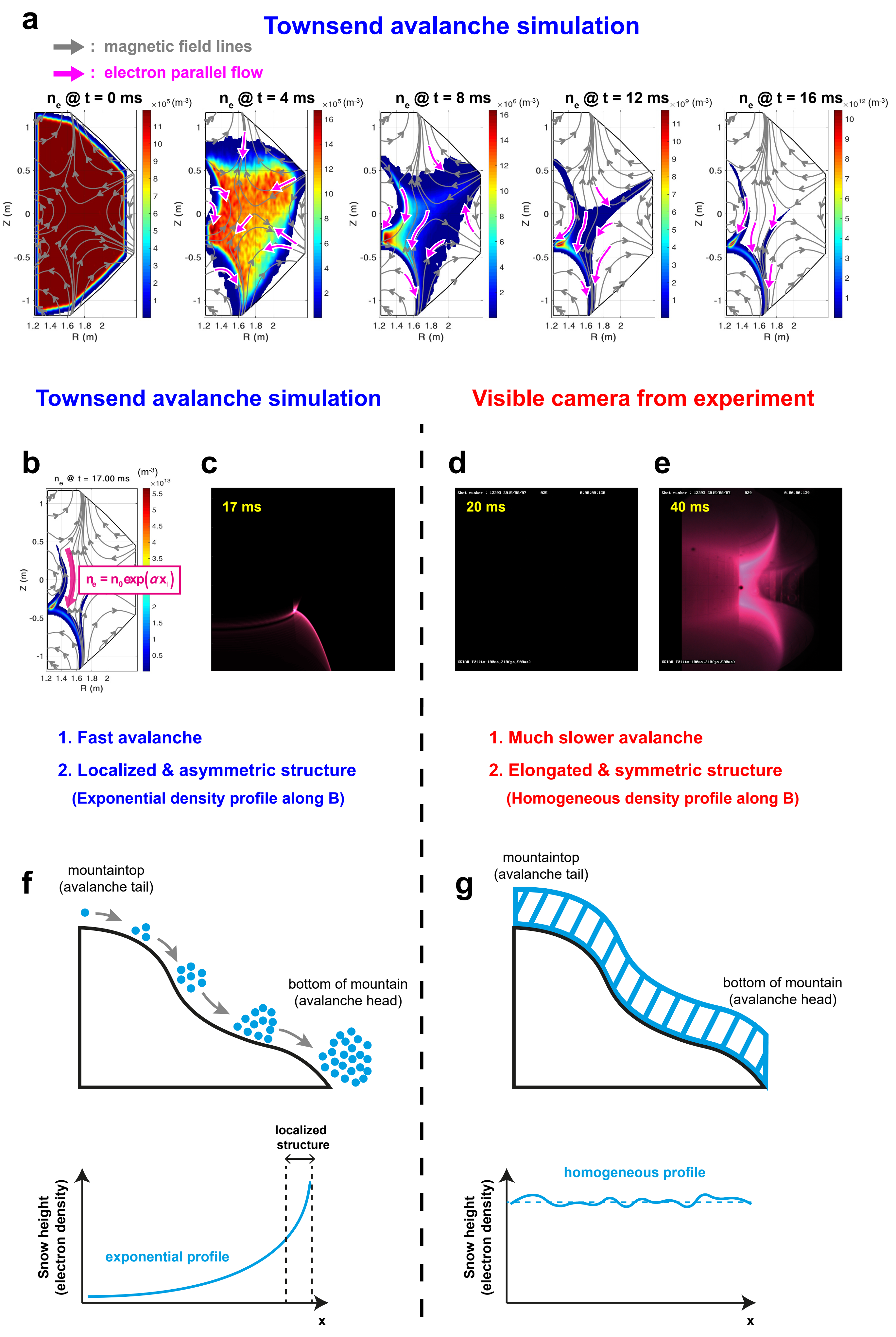

Supplementary Figure 3 | Comparison between Townsend avalanche simulation and experiments.

a, Temporal evolution of electron density in the Townsend avalanche simulation. The grey arrows and magenta arrows indicate the magnetic field lines and the electron parallel flows, respectively. **b**, Snapshot of the plasma density at t = 17 ms. The plasma density structure has the exponential profile along the magnetic field line. **c**, Synthetic diagnostic of Balmer-α emission from the Townsend simulation. The emission structure is very localized and asymmetric due to the exponential profile of the plasma density. **d** and **e** are the visible camera image of the Balmer-α emission from experiments. d, No signal is observed at t = 20 ms in contrast to the Townsend's prediction. e, The elongated and symmetric emission structure along the magnetic field lines is observed at t = 40 ms. These indicate that actual electron avalanche is much slower than Townsend's prediction, and the plasma density is homogenous along the magnetic field line. **f**, Principle of the normal snow avalanche that has an exponential height profile along the mountain slope. **g**, The actual electron avalanche of the ohmic breakdown can be compared with the abnormal snow avalanche that has a homogeneous snow height from the top of the mountain to the bottom of the mountain.

Supplementary Figure 4

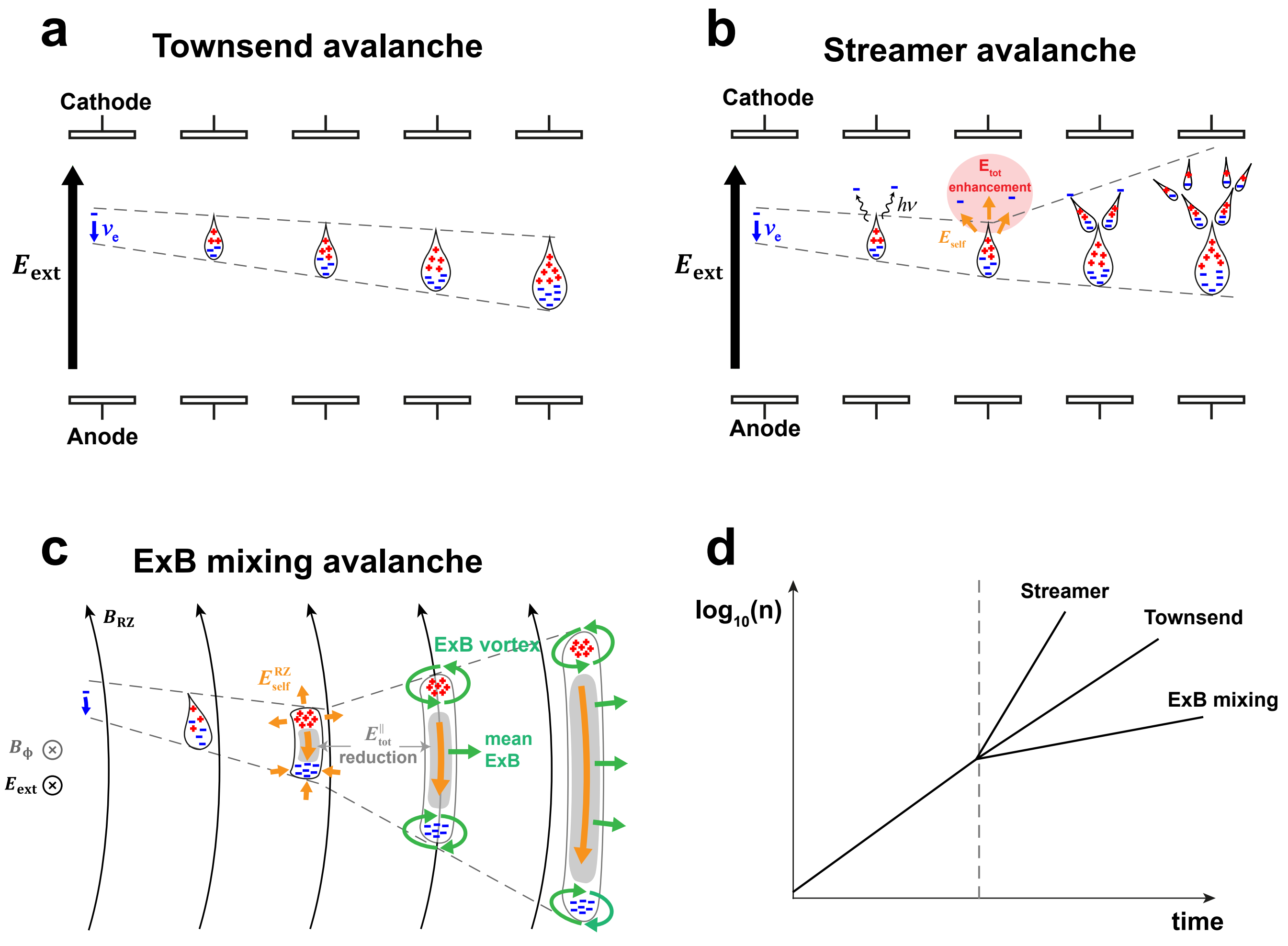

Supplementary Figure 4 | Schematic diagrams of three different types of gas breakdown. **a**, Townsend avalanche grows exponentially due to the dominant external electric fields. **b**, Streamer avalanche is much faster than the Townsend avalanche, because the seed electrons produced by photoionization at the avalanche tail make the secondary avalanches due to the self-electric fields. **c**, ExB mixing avalanche is much slower than other avalanches because the self-electric fields cancel the external electric fields in the main plasma region. The turbulent mixing by ExB vortices at the plasma edge rapidly diffuses the plasma along $\mathbf{B}_{RZ}$, and the mean ExB moves the plasma across $\mathbf{B}_{RZ}$. **d**, Temporal evolution of the plasma density in log-scale for each type of the avalanche.

Supplementary Figure 5

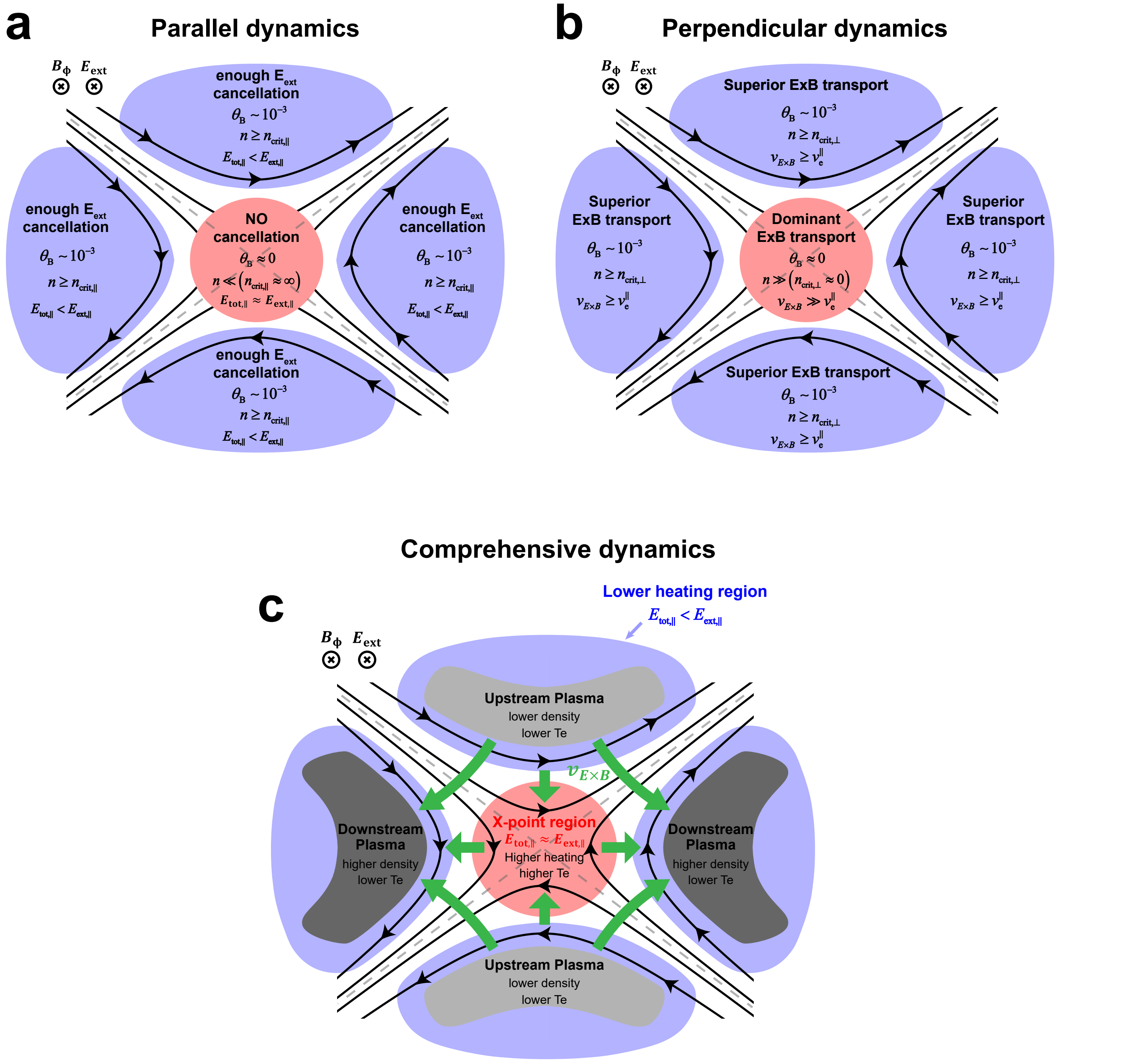

Supplementary Figure 5 | Topology analysis of quadrupole EM structure regarding X-point. Topology analysis of the EM structure regarding **a**, parallel dynamics and **b**, perpendicular dynamics. The X-point region (red shaded region) has near zero pitch angle $(\theta_B \to 0)$ that results in an infinite parallel critical density $(n_{crit,||} \propto \cot^2\theta_B \to \infty)$ and an infinitesimal perpendicular critical density $(n_{crit,\perp} \propto \tan^2\theta_B \to 0)$, respectively. Other regions (blue shaded region) have moderate parallel and critical perpendicular densities corresponding to the pitch angle $(\theta_B \sim 10^{-3})$. **c**, Topology analysis can predict overall structure and behaviors of the plasma evolution by considering the mean ExB flows and the inhomogeneous heating power.

Supplementary Figure 6

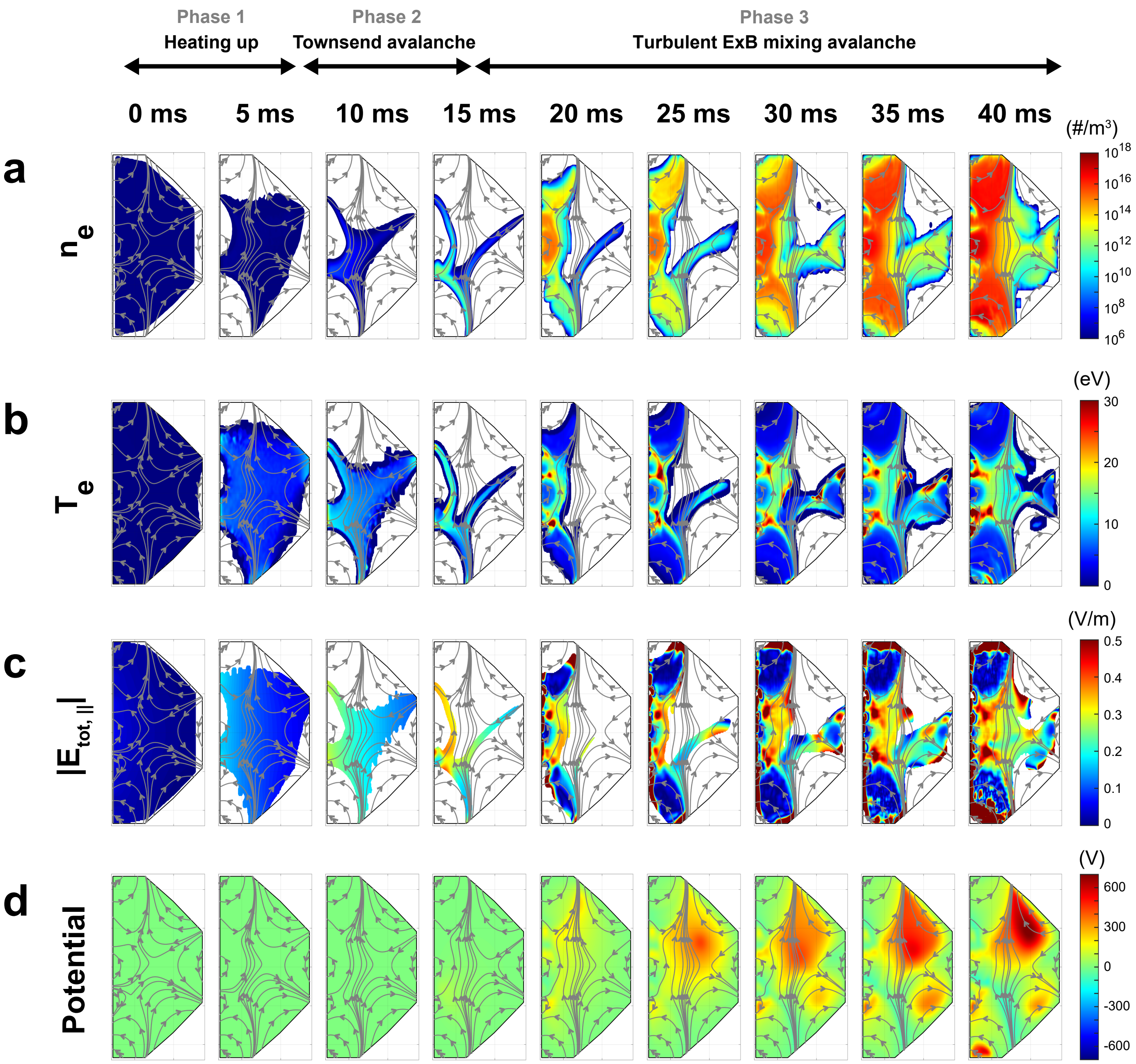

Supplementary Figure 6 | 2D snapshots of particle simulation results of KSTAR reference breakdown scenario.

2D snapshots in the RZ plane of **a**, electron density in log-scale, **b**, electron temperature, **c**, total parallel electric fields $E_{tot,||}$, and **d**, electrostatic plasma potential in the RZ plane from 0 to 40 ms. In phase 3, as the plasma density exceeds the critical density, the plamsa potential structure built up in the device produces strong self-electric fields. These self-electric fields significantly change the total parallel electric fields, and subsequently, the electron temperature at X-point regions becomes higher than that of other regions. The newly enhanced perpendicular transports via ExB drift cause the homogeneous electron density along the magnetic field lines.

Supplementary Figure 7

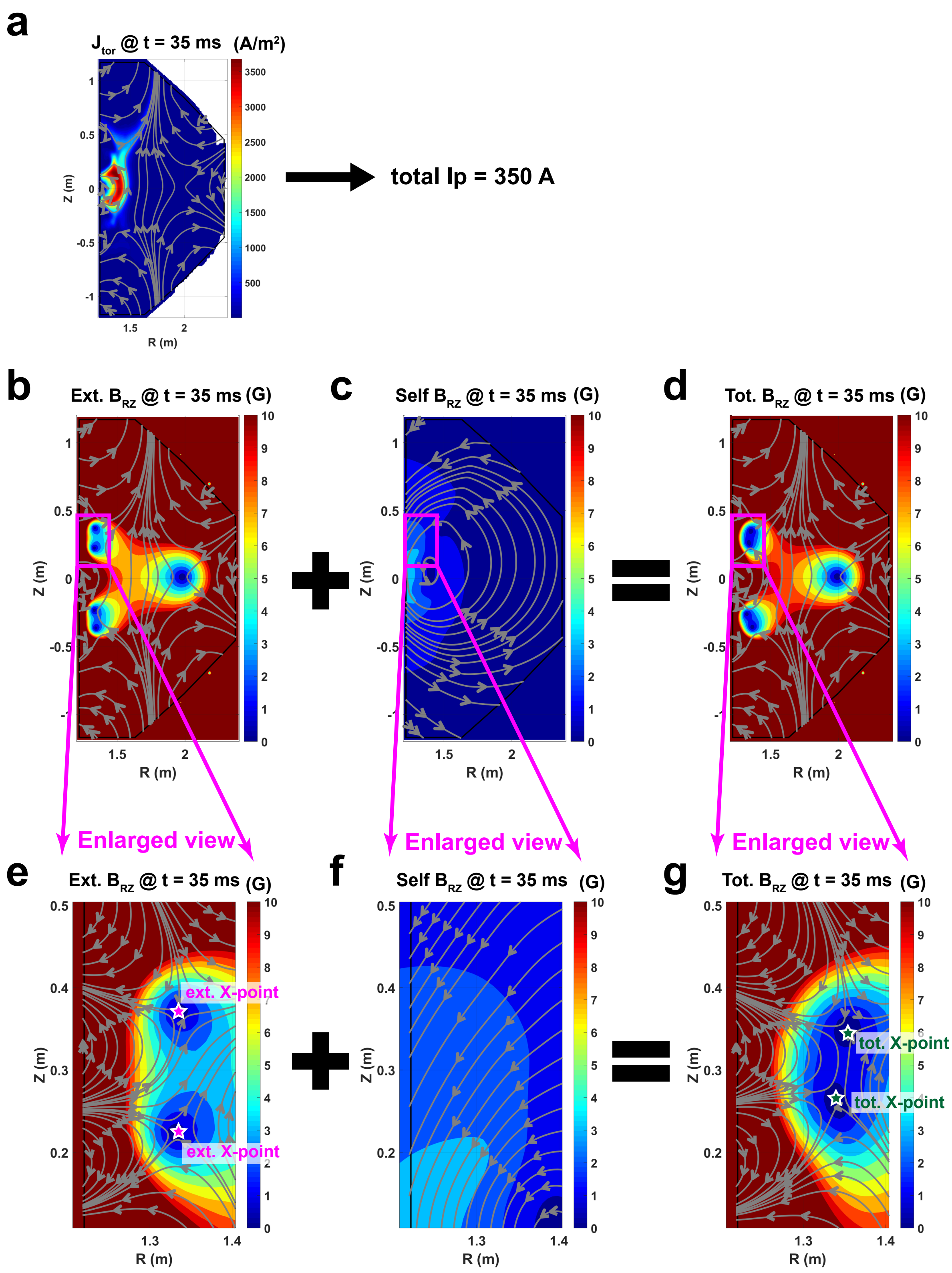

Supplementary Figure 7 | Toroidal plasma currents and poloidal self-magnetic fields. **a**, Toroidal plasma current density in the RZ plane at t = 35 ms of the KSTAR ohmic breakdown simulation. **b**, Externally driven poloidal magnetic fields $\mathbf{B}_{\mathrm{ext}}^{RZ}$, **c**, self-magnetic fields $\mathbf{B}_{\mathrm{self}}^{RZ}$ produced by the toroidal plasma currents, and **d**, total poloidal magnetic fields $\mathbf{B}_{\mathrm{tot}}^{RZ}=\mathbf{B}_{\mathrm{ext}}^{RZ}+\mathbf{B}_{\mathrm{self}}^{RZ}$. Enlarged views at inboard side for **e**, external, **f**, self, and **g**, total magnetic fields.

Supplementary Figure 8

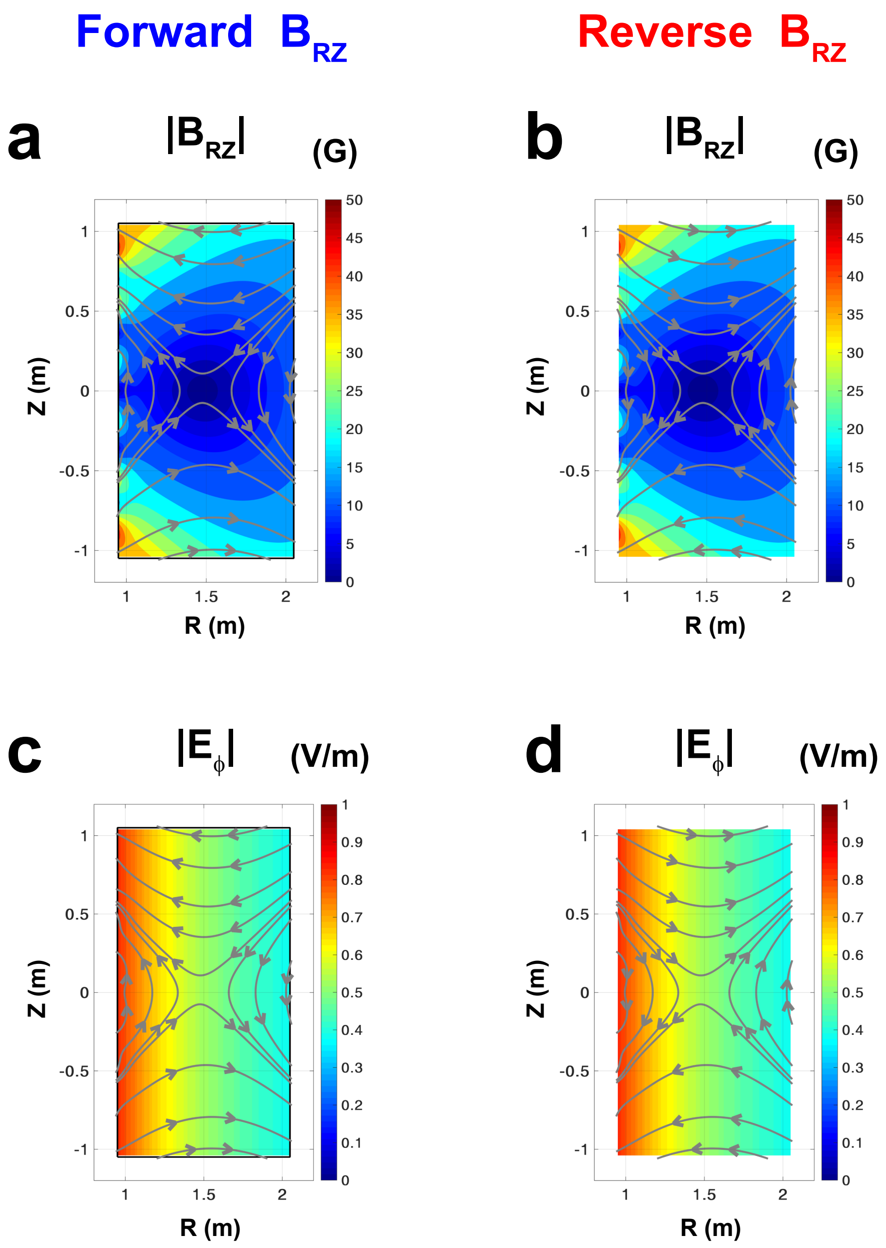

Supplementary Figure 8 | Electromagnetic structures of the artificial single X-point scenarios.

Two cases, forward and reverse $\mathbf{B}_{RZ}$, that have the same magnitudes but opposite direction of the vertical magnetic fields $\mathbf{B}_{RZ}$ are compared. **a** and **b** are magnitudes of the vertical magnetic fields of the forward and the reverse $\mathbf{B}_{RZ}$ case, respectively. **c** and **d** are magnitudes of the toroidal electric fields for the two cases. Grey lines and arrows indicate the magnetic field lines.

Supplementary Figure 9

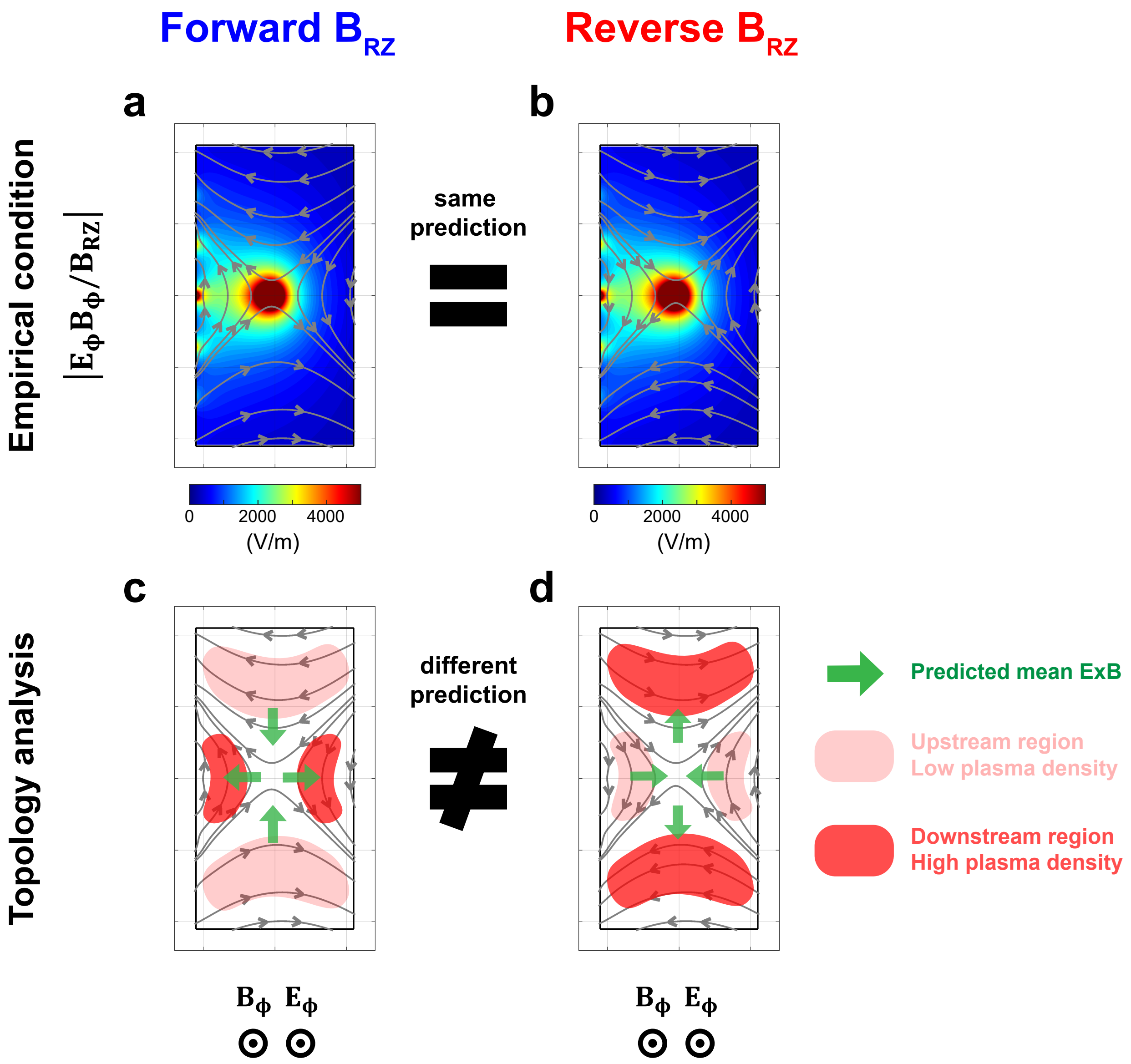

Supplementary Figure 9 | Traditional empirical condition and novel topology analysis method. Traditional empirical condition (**a**, **b**) and our novel topology analysis method (**c**, **d**) are compared for the forward and the reverse $\mathbf{B}_{RZ}$ scenario. The empirical condition for the two cases gives the same predictions that the plasma will be produced at the X-point region because the magnitudes of the EM fields are exactly same in the two cases. On the other hand, the topology analysis method predicts that the two cases have different mean ExB flows (green arrows) due to their opposite $\mathbf{B}_{RZ}$ directions. Accordingly, the forward $\mathbf{B}_{RZ}$ case has two downstream regions at the left and the right side of the X-point region. The reverse $\mathbf{B}_{RZ}$ case has two downstream regions at the upper and the lower side of the X-point region. The plasma density of the downstream regions is higher than that of the upstream regions. These predictions from the topology analysis method of the external EM fields agree well with the simulation results (Supplementary Figure 10c, d).

Supplementary Figure 10

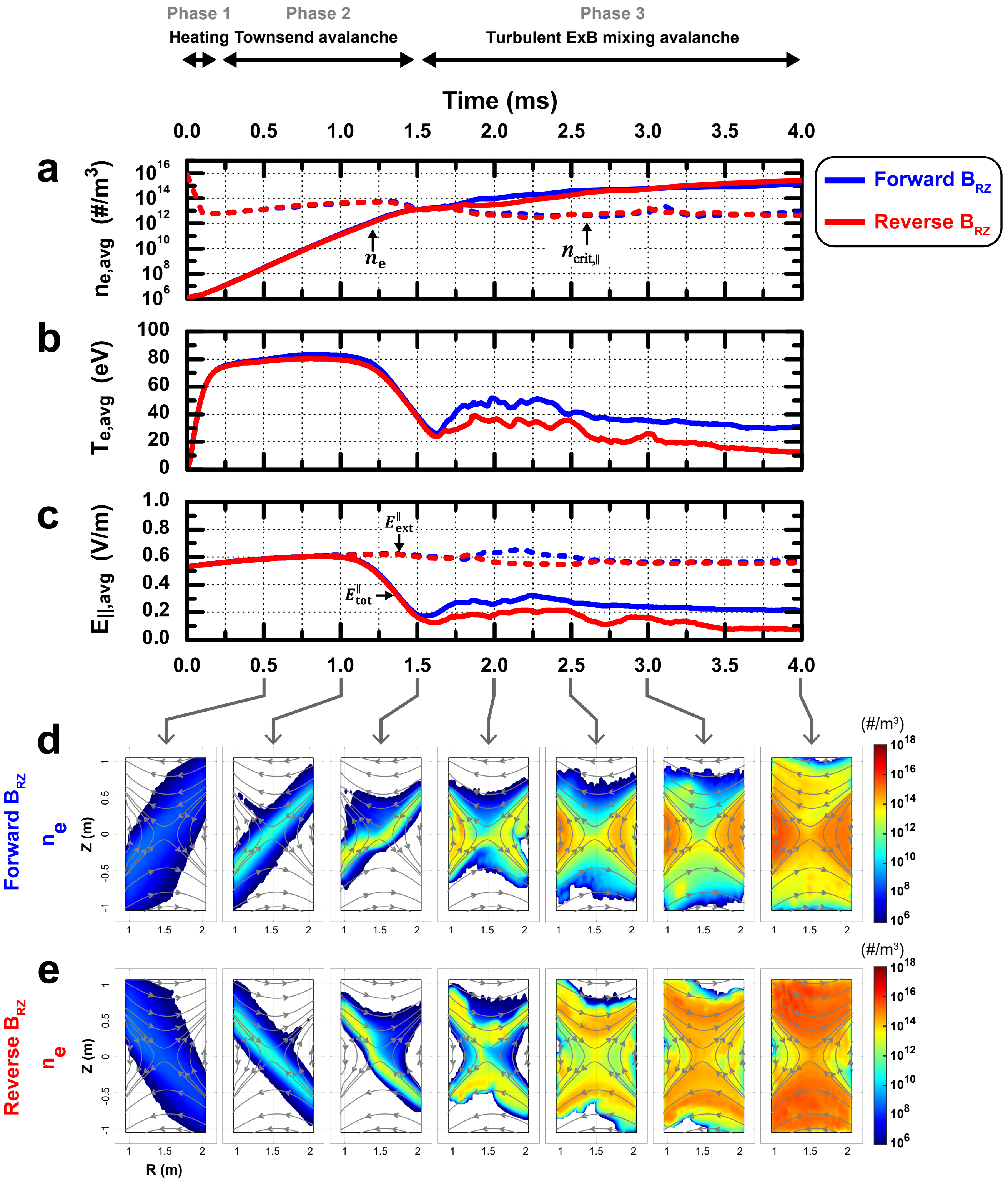

Supplementary Figure 10 | Particle simulation results of artificial single X-point scenarios. Particle simulation results for the forward (blue) and the reverse (red) $\mathbf{B}_{RZ}$ case. Temporal evolutions of **a**, average electron density (solid) and critical parallel density (dot) in log-scales, **b**, average electron temperatures, and **c**, average magnitudes of parallel components of the external electric fields (dot) and total electric fields (solid). Two-dimensional snapshots of the log-scale electron densities of the two cases are compared in **d** and **e**. The forward $\mathbf{B}_{RZ}$ case has two high-density regions at the left and the right side of the X-point. The reverse $\mathbf{B}_{RZ}$ case has two high-density regions at the upper and the lower side of the X-point region. These results agree well with the predictions from the topology analysis method (Supplementary Figure 9c, d).
