# Supplementary material for: Evidence of a turbulent ExB mixing avalanche mechanism of gas breakdown in strongly magnetized systems
Source: Nat Commun. 2018 Aug 30;9:3523. doi: 10.1038/s41467-018-05839-5 (PMC6117305; doi:10.1038/s41467-018-05839-5)
Supplement: Supplementary file 2 — Description of Additional Supplementary Files [file 41467_2018_5839_MOESM2_ESM.pdf]

Description of Additional Supplementary Files

File Name: Supplementary Movie 1

Description: Comparison between two simulation results of the KSTAR ohmic breakdown scenario with and without considering the self-electric fields. (Upper) Averaged log-scale electron density. (Lower) Log-scale electron density in the RZ plane without (Left) and with (Right) considering the self-electric fields. The magenta arrows indicate the directions and relative magnitudes of the total electron flow velocities.

File Name: Supplementary Movie 2

Description: Fluctuating nature of the ohmic breakdown plasma. 2D snapshots of the ohmic breakdown plasma: (1) linear-scale electron density, (2) charge-density, (3) vorticity, (4) parallel component of the total electric field, and (5) its temporal average.

File Name: Supplementary Movie 3

Description: (Left) Color contour is the charge density and green arrows indicate the directions and magnitudes of total electron flow velocities that are sum of the parallel electron flows and ExB flows. (Right) Color contour is the electron density and magenta z-surfaces are the electron losses at the wall.

File Name: Supplementary Movie 4

Description: Detailed simulation results of the KSTAR ohmic breakdown scenario. (Upper Left) Linear-scale electron density, (Upper Middle) Log-scale electron density, (Upper Right) Log-scale ion density, (Lower Left) Electron temperature, (Lower Middle) Absolute magnitudes of the total parallel electric fields, and (Lower Right) Plasma potential.

File Name: Supplementary Movie 5

Description: Comparison between two simulation results of artificial simple X-point scenarios. Temporal evolutions of log-scale electron densities of (Left) Forward $\mathbf{B}_{RZ}$ case and (Right) Reversed $\mathbf{B}_{RZ}$ case.
